# Crosslinked-hybrid nanoparticle embedded in thermogel for sustained co-delivery to inner ear

**DOI:** 10.1186/s12951-024-02686-z

**Published:** 2024-08-13

**Authors:** Neeraj S. Thakur, Iulia Rus, Aidan Herbert, Marisa Zallocchi, Brototi Chakrabarty, Aditya D. Joshi, Joshua Lomeo, Vibhuti Agrahari

**Affiliations:** 1 Department of Pharmaceutical Sciences, University of Oklahoma Health Sciences Center, 1110 North Stonewall Avenue, Oklahoma City, OK 73117 USA; 2 DigiM Solution LLC, 500 West Cummings Park, Suite 3650, Woburn, MA 01801 USA; 3 Department of Biomedical Sciences, Creighton University School of Medicine, 2500 California Plaza, Omaha, NE 68178 USA

**Keywords:** Artificial intelligence image analysis, Central composite design, Deep learning model, Drug-induced-ototoxicty, Hearing loss, Local drug delivery, Long-term drug delivery, Otoprotectants, Redox homeostasis, Quality-by-design approach, Zebrafish model

## Abstract

Treatment-induced ototoxicity and accompanying hearing loss are a great concern associated with chemotherapeutic or antibiotic drug regimens. Thus, prophylactic cure or early treatment is desirable by local delivery to the inner ear. In this study, we examined a novel way of intratympanically delivered sustained nanoformulation by using crosslinked hybrid nanoparticle (cHy-NPs) in a thermoresponsive hydrogel i.e. thermogel that can potentially provide a safe and effective treatment towards the treatment-induced or drug-induced ototoxicity. The prophylactic treatment of the ototoxicity can be achieved by using two therapeutic molecules, Flunarizine (FL: T-type calcium channel blocker) and Honokiol (HK: antioxidant) co-encapsulated in the same delivery system. Here we investigated, FL and HK as cytoprotective molecules against cisplatin-induced toxic effects in the House Ear Institute - Organ of Corti 1 (HEI-OC1) cells and in vivo assessments on the neuromast hair cell protection in the zebrafish lateral line. We observed that cytotoxic protective effect can be enhanced by using FL and HK in combination and developing a robust drug delivery formulation. Therefore, FL-and HK-loaded crosslinked hybrid nanoparticles (FL-cHy-NPs and HK-cHy-NPs) were synthesized using a quality-by-design approach (QbD) in which design of experiment-central composite design (DoE-CCD) following the standard least-square model was used for nanoformulation optimization. The physicochemical characterization of FL and HK loaded-NPs suggested the successful synthesis of spherical NPs with polydispersity index < 0.3, drugs encapsulation (> 75%), drugs loading (~ 10%), stability (> 2 months) in the neutral solution, and appropriate cryoprotectant selection. We assessed caspase 3/7 apopototic pathway in vitro that showed significantly reduced signals of caspase 3/7 activation after the FL-cHy-NPs and HK-cHy-NPs (alone or in combination) compared to the *Cis*Pt. The final formulation i.e. crosslinked-hybrid-nanoparticle-embedded-in-thermogel was developed by incorporating drug-loaded cHy-NPs in poloxamer-407, poloxamer-188, and carbomer-940-based hydrogel. A combination of artificial intelligence (AI)-based qualitative and quantitative image analysis determined the particle size and distribution throughout the visible segment. The developed formulation was able to release the FL and HK for at least a month. Overall, a highly stable nanoformulation was successfully developed for combating treatment-induced or drug-induced ototoxicity via local administration to the inner ear.

**Graphical Abstract:**

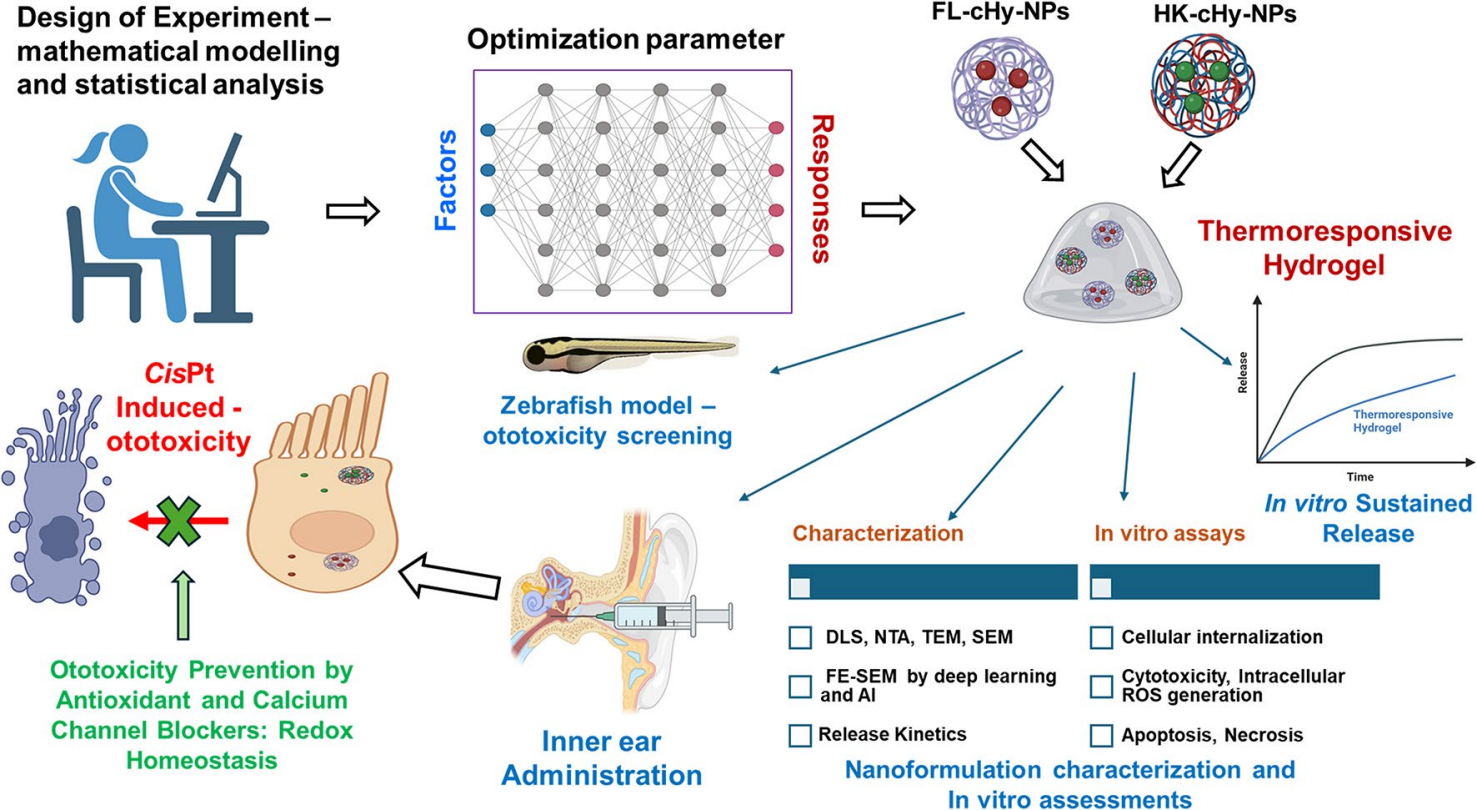

**Supplementary Information:**

The online version contains supplementary material available at 10.1186/s12951-024-02686-z.

## Introduction

Treatment-induced ototoxicity has been reported [1–3] for different categories of drugs such as aminoglycosides, vancomycin, macrolides, loop diuretics, etc. Although the ototoxicity of drug categories such as loop diuretics, macrolide antibiotics, and quinine is reported to be reversible when treatment is stopped, a few cases have been reported for occasional irreversible hearing loss (HL) [1, 4]. However, platinum drugs, especially cisplatin, and aminoglycosides such as kanamycin, cause irreversible damage to the outer hair cells (OHCs) and inner ear hair cells (IHCs) of the cochlea. Thus, hearing impairment or permanent hearing loss significantly affects patients quality of life. Treatment-induced hearing loss (TIHL) lead- to considerable follow-up costs estimated $300,000 per adult who acquired hearing impairment due to ototoxic medication [4]. Considering ototoxicity as well-known obstacle to several therapeutic classes of drugs, a prophylactic cure or early treatment is crucial for protecting IHCs and OHCs. [5, 6]. The mechanism involves accumulation of drugs in the cochlea, oxidative stress, apoptosis, and inflammation, all of which can lead to cell death and hearing loss [7]. To mitigate ototoxicity, protective strategies such as otoprotective agents and careful monitoring of patients’ hearing can be developed [5]. For instance, many cytoprotective molecules have been investigated as cytoprotective agents against *Cis*Pt-induced ototoxicity (CIO) such as Amifostine [8], Sodium Thiosulfate (STS) [9, 10], Dexamethasone [11, 12], N-acetylcysteine [13–15], Ebselen [16], Flunarizine [17], Agmatine [18], Honokiol [19], and Allicin [20] are few [21, 22]. Recently, the FDA approved sodium thiosulfate IV for prevention in children [23], however, no treatments are available to prevent or reverse CIO in adults. Moreover, patient-centric compliance with the administration of any molecule still requires significant research and development. Therefore, developing effective formulations for local delivery to the inner ear is one of the challenges in preventing treatment-induced ototoxicity and hearing loss due to ototoxic medications.

Flunarizine (FL), a ‘T-type’ calcium channel blocker, has been studied for its potential to mitigate cisplatin-induced cell cytotoxicity and ototoxicity. However, this effect was not mediated by the modulation of intracellular calcium levels [24]. So et al. reported the cytoprotective effect of FL against CIO in the House Ear Institute - Organ of Corti 1 (HEI-OC1) cells through the downregulation of NF-κB via Nrf2/HO-1 activation which results in reduced pro-inflammatory cytokine production [17, 25]. Honokiol (HK), a natural compound found in the Magnolia tree, has been investigated for its potential protective effects against CIO. Its antioxidant properties may help reduce reactive oxygen species (ROS) production, protecting the inner ear from oxidative damage. Its anti-inflammatory properties may mitigate the inflammatory response, potentially reducing cisplatin-induced damage. Mechanistically, it has been reported that it activates sirtuin 3 (SIRT-3), a mitochondrial deacetylase that is responsible for ROS detoxification [19]. In addition, combination therapy with other molecules may be explored to obtain promising cytoprotective effects against treatment-induced or drug-induced ototoxicity and associated hearing loss. Notably, after clinical safety and efficacy validation in humans, the combination of FL and HK based formulations could be a promising strategy for reducing cisplatin-induced cytotoxicity and ototoxicity.

Design of Experiments (DoE) is a systematic approach to planning, conducting, and analyzing experiments to optimize processes, products, or systems. It is a structured approach based on mathematical and statistical analysis and is widely used in fields such as science, engineering, manufacturing, and quality control [26, 27]. The key steps include defining the objective, identifying factors and levels, selecting an appropriate experimental design, conducting experiments, analyzing data, optimizing, validating, drawing conclusions, and providing recommendations [28]. Common designs include full factorial design, fractional factorial design, response surface design, Taguchi design, randomized complete block design, and randomization [29]. Central Composite Design (CCD) is a widely used experimental design in DoE for efficient exploration of a wide range of factor space, optimization, robustness analysis, handling of quadratic effects, reduced experimental effort, flexibility, and statistical efficacy. It is often used for response surface modeling and identifying optimal conditions for achieving desired responses. CCD also assesses the robustness of a system or process by evaluating its performance across different factor levels, ensuring stability and reliability in real-world applications. Its systematic structure, including randomization and replication of experimental runs, minimizes the impact of uncontrolled factors or random errors, leading to more reliable results [30–32]. Therefore, CCD can be considered as an essential tool in formulation development experimentation, and process optimization. The presented method highlights operating DoE-CCD as a supplementary tool for the design of efficient drug delivery system. The standard least square fit model was applied to optimize the formulation factors for the synthesis of FL-and HK-loaded crosslinked hybrid nanoparticles (FL-cHy-NPs and HK-cHy-NPs). After running the model in the software, the model fit for each response (particle size, polydispersity index, encapsulation efficiency, and loading efficiency) was evaluated by various analyses such as ANOVA, Lack of Fit, Actual vs. predicted plots, Scatter Index (SI), Residual Vs predicted plots and studentized plots. Further, the combination of effects on each response was determined by contour plots. Lastly, the optimized values of the factors for the desired synthesis of FL-cHy-NPs and HK-cHy-NPs were determined using prediction profiler graphs.

The objective of our investigation was to protect hair cells in the inner ear by fabricating an effective biomaterial-based drug delivery system for TIHL and drug-induced ototoxicity. *Cis*Pt was used as a model cytotoxic agent for this study. Herein, highly stable FL- and HK-loaded crosslinked hybrid nanoparticles (cHy-NPs) were synthesized using the DoE approach by applying a CCD model. The physicochemical characterization and stability studies of synthesized NPs were performed and there in vitro cytoprotective ability against *Cis*Pt-induced toxicity was subsequently determined. in HEI-OC1 cells. The zebrafish model was utilized as in vivo model to test the therapeutic potential of FL- and HK- cHy-NPs against *Cis*Pt-induced toxicity. Finally, a thermoresponsive hydrogel formulation was developed by incorporating FL-cHy-NPs and HK-cHy-NPs in a blend of Poloxamer 407, Poloxamer 188, and Crabomer 940-based hydrogels for sustained drug release and the release kinetics were determined at 25 and 37 °C. Furthermore, a combination of artificial intelligence (AI)-based image analytical techniques was used to determine the particle location within the scene, including a deep learning semantic segmentation model. Our study identified a novel delivery system ‘cHy-NPs embedded in thermogel’, to prolong the residence time at the target organ by local application for treatment-induced or drug-induced ototoxicty.

## Materials and methods

### Materials

Flunarizine (FL, TCI), cis-diammineplatinum(II) dichloride (*Cis*Pt, TCI), chlorpromazine (CPZ, TCI), amiloride (AML, TCI), methyl-β-cyclodextrin (MβCD, TCI), genistein (GNT, TCI), triethylamine (TEA, Fisher Chemical), acetonitrile (ACN, Fischer Chemical), methanol (Fisher Chemical), dichloromethane (DCM, Fischer Chemical), propidium iodide (PI, MP Biomedicals), coumarin-6 (C6, Thermo Fischer), 10,12-Pentacosadiynoic acid (PCDA; TCI), HOECHST-33342 (Tocris Biosciences), 2’,7’-dichlorofluorescein 3’,6’-diacetate (DCFH_2_-DA, Thermo Fisher), poloxamer-188 (P-188, Alfa Aesar), hydrochloric acid (HCl, Fisher Chemicals) and carbomer-940 (C-940, Spectrum) were purchased from Fischer Scientific, USA. Poloxamer-407 (P-407), CaspaTag™ In Situ Caspase-3/7 Detection Kit, and trypsin-EDTA solution (0.25%) were purchased from Millipore Sigma, USA. Polypropylene sulfide-methoxy polyethyleneglycol-2000 (PPS-mPEG_2000_) was synthesized in-house [33]. Honokiol (HK, > 98%) was purchased from MCS Formulas B. V., Netherlands. The cell culture media, reagents, and phosphate buffered saline (PBS) were procured from Millipore Sigma, USA. MitoSOX™ Cat: M36008, assay kit was received as a kind gift from Invitrogen, USA.

### HPLC method development of flunarizine (FL) and honokiol (HK)

The qualitative and quantitative analytical methods of the FL and HK were developed on a high-performance liquid chromatography (HPLC) system utilizing a previous protocol with some modifications [34, 35]. The HPLC system (1260 Infinity, Agilent Technologies, Santa Clara, CA, USA) equipped with a VWD (UV-vis detector) was utilized for the method development. Different concentrations of FL and HK (0.048828–100 µg/mL) were prepared separately from the respective stock solutions (2 mM) in methanol. The samples were run in HPLC according to the International Conference on Harmonization (ICH) protocol for qualitative and quantitative method validation (3 samples of each concentration per day for three consecutive days) utilizing the method parameters listed in Table S1 and representative chromatograms Figure S1. Validation calculations were performed using the smallest five concentrations (0.048828–0.78125 µg/mL; see Tables S2–S3 Figures S2-S3). The standard curves for each data set were drawn and the equations were determined. The limit of detection (LOD) and limit of quantification (LOQ) were further determined using the slopes of the standard equations by following formulas [36]:

1 $$\text{L}\text{O}\text{D}=\frac{3.3\sigma }{S}$$

2 $$\text{L}\text{O}\text{Q}=\frac{10\sigma }{S}$$

where, $\sigma$ is the standard deviation of the slope, and $S$ is is the slope (mean) of the graph.

### Preparation of FL-cHy-PCDA-PPS-mPEG_2000_-NPs (FL-cHy-NPs) and HK-cHy-PCDA-PPS-mPEG_2000_-NPs (HK-cHy-NPs)

For the final synthesis of FL-cHy-NPs and HK-cHy-NPs, PPS-mPEG_2000_ (14 and 15 mg for FL and HK, respectively), PCDA (50% w/w of PPS-mPEG_2000_) and FL or HK (12.5% w/w of PPS-mPEG_2000_ and PCDA) were dissolved in 1 mL of DCM in a microcentrifuge tube. In a 15 mL centrifuge tube containing 2% PVA solution (4 mL), this mixture was mixed under vortexing and then -sonicated using a 20 kHz ultrasonicator (Fischer Scientific, USA) for 20 s (three times). The colloidal emulsion was then transferred to a small beaker containing 10 mL of 0.3% PVA solution and stirred at 600 rpm for 2 h. The colloidal solution of NPs then centrifuged at 15,000 rpm for 10 min and after discarding the supernatant, the pellet was resuspended in 5 mL deionized (DI) water. The suspended colloidal NP solution was kept under UV light (254 nm) for crosslinking and obtaining cHy-NPs. Before the final synthesis of cHy-NPs, various synthesis parameters were optimized by design of experiment (DoE) approach using central composite design (CCD) (JMP^®^ Pro 16, SAS, NC, USA) to obtain favorable size, polydispersity index (PDI), encapsulation efficiency (EE, %), and loading efficiency (Ld, %) [37]. Finally, synthesized colloidal NP solutions were lyophilized using sucrose (2.5% w/v) as a cryoprotectant (for details see  “Storage stability and freeze drying” section).

### Design of experiment-central composite design (DoE-CCD)

With JMP^®^ pro 16 software, the response surface design window was opened by clicking on “DOE”>“Classical”>“Response Surface Design”. Four responses, (i) particle size (nm), (ii) polydispersity index (PDI), (iii) encapsulation efficiency (EE %), and (iv) loading efficiency (Ld %) were added in the window. The goal of the particle size and PDI responses were kept “minimize” while the goal for EE (%) and Ld (%) were kept “maximize”. The upper and lower limits were also set up in the table. In the factors section, three continuous factors were added as (i) PPS-mPEG_2000_ (mg/mL), (ii) PCDA (% w/w of PPS-mPEG_2000_), and (iii) drugs (FL or HK, % w/w of PPS-mPEG_2000_ and PCDA). The values (-1 and + 1) for PPS-mPEG_2000_ (5 and 20), PCDA (10 and 100), and drugs (5 and 20) were added (Figure S4). After all the responses, the central composite design was selected. In the “display and modify design” section, the axial value was kept ‘1.000’, ‘On Face’ was selected and the run order selected ‘Keep the same’ and then clicked on ‘Make Table’ (Figure S5). The CCD design table was generated (Figure S6). As detailed in SI Table S4 the reactions were performed accordingly (following  “Preparation of FL-cHy-PCDA-PPS-mPEG” section) for each run. The particle size and PDI value of cHy-NPs in each sample were determined by dynamic light scattering (DLS). The EE (%) and Ld (%) of drugs in each sample were determined using the following formulas:

3 $$\text{E}\text{E}\left(\text{\%}\right)=\frac{\text{C}}{{C}_{0}}\times 100$$

4 $$\text{D}\text{L}\left(\text{\%}\right)=\frac{\text{C}}{{P}_{0}{+C}_{o}}\times 100$$

where, $\text{C}$ is the concentration of the drug into the cHy-NPs, ${C}_{0}$ is the initial drug concentration, ${P}_{0}$ is the concentration of the added polymer.

All acquired data were put in the CCD table (Tables S5 and S6), then by clicking at ‘Model’ (green arrow), “fit model window” was open. In the ‘Pick Role Variable’ section, all four responses were added to ‘Y’ section. The personality was settled as ‘Standard Least Square’ and emphasis was set as ‘Effect Screening’, then the model was run by hitting ‘run’ button (Figure S7). In the least square fit section, the non-significant factors were removed from the ‘Effect Summary’ table one by one (Figure S8). The analysis of variance (ANOVA), Lack of Fit, Actual by Predicted Plot, Residual by Predicted Plot, and Studentized Residuals for each response were obtained from least squares fit model to evaluate whether the model fit well (Figure S9). The scatter index (SI%) of each response was determined using the root mean square error (RMSE) and mean response ($\stackrel{-}{X}$) by applying the following formula:

5 $$SI\left(\%\right)=\frac{RMSE}{\stackrel{-}{X}}\times 100$$

After confirming good fit, the predicted formula column for each response was added to the response table by clicking on ‘red down arrow’ (beside each response)>‘Save Columns’>‘Prediction Formula’ (Figure S10). The contour profiler used to determine the optimized factor for the synthesis of FL-cHy-NPs and HK-cHy-NPs were generated using the prediction formula. To prepare the contour profiler, clicked on ‘Graph’> ‘Contour Profiler’(Figure S11) then the prediction formula for each response was added to the ‘Y, prediction formula’ section and clicked ‘OK’ (Figure S12). The effect of a combination of two factors at a time was determined to obtain the desired response. The optimal factor concentrations for a desired response were then calculated using the prediction profiler graphs in the least square fit window (Figure S13).

### Characterization of FL-cHy-NPs and HK-cHy-NPs

The size and polydispersity (PDI) of the synthesized cHy-NPs were determined using dynamic light scattering (DLS). Samples were prepared by adding 10 µL of freshly prepared cHy-NPs to 990 µL of DI water and analyzed using a DLS instrument equipped with a 635 nm laser (Brookhaven Instruments, Holtsville, NY, USA). Furthermore, the morphology and size were confirmed using transmission electron microscopy (TEM) analysis. Briefly, diluted colloidal solutions (10 µL) were dropped on the carbon-coated copper grid (200 mesh). After 5 min, the grids were washed twiced with DI water (30 s) and 10 µL staining solution (uranyl acetate, 2% w/v) was dropped on the grids. After 1 min, the grids were washed again with deionized water 2 times and air-dried for 10 min. The samples were analyzed using TEM (Hitachi H-7600, Hitachi, Japan) at 80 kV and the images were captured using NANOSPRT12 camera with 800 (ms) × 4 drift frames exposed for normal contrast.

### Storage stability and freeze drying

The storage stability of the developed FL-cHy-NPs and HK-cHy-NPs was determined by keeping them at 4 °C in the solution for at least 60 days. The size and PDI values for stored samples at 0, 7, 21, 36, and 60 day intervals were recorded using DLS. The effects of various cryoprotectants (glucose, mannitol, sucrose, and trehalose) were determined. Momentarily, different amounts (1, 2.5, 5, 7.5, and 10% w/v) of cryoprotectants were added to the colloidal solution of cHy-NPs and lyophilized using Triad Benchtop Freeze Dryer (Labconco, Kansas City, MO, USA). Freeze-dried samples were reconstituted in DI water, after which the size and PDI of the samples were recorded using DLS.

### Cellular internalization study

First, blank cHy-NPs (cHy-NPs, without FL or HK encapsulation) were prepared as described above (Preparation of FL-cHy-PCDA-PPS-mPEG-NPs section). In, 1 mL colloidal solution of cHy-NPs, 1 mg coumarin-6 (C6) was added and the mixture was stirred at 200 rpm overnight at room temperature. The NP solution was then centrifuged at 2000 rpm for 5 min to remove unloaded C-6. The supernatant containing C6-cHy-NPs was transferred to a fresh microcentrifuge tube. HEI-OC1 cells were seeded in 96 well plate (10,000 cells per well) overnight. After washing with fresh growth media, the media (100 µL) containing 40 µg/mL chlorpromazine (CPZ), methyl-β-cyclodextrin (MβCD), genistein (GNT), or amiloride (AML) was added to separate columns (2 columns kept untreated as a control group). After 1 h of incubation at 33 °C (5% CO_2_), 2.5 µL of C6-cHy-NPs (10x diluted with media) was added to each well. The plate was incubated for 4 h, after which the cells were washed with PBS. After adding fresh PBS, the fluorescence intensity of each well was recorded using a multi-well plate fluorescence spectrophotometer (Synergy 2, BioTek, USA) at λex/em 460/505. In another experiment to confirm the effect of various inhibitors, the cells (20,000 per well) were seeded overnight, and incubated with different concentrations (0, 2.5, 5, 10, 20, 30, 40, 50, and 75 µg/mL) of MβCD, GNT, or AML. After treatment, incubation, and washing, the fluorescence intensity of each well was recorded as described above. Furthermore, to study the time of cellular internalization the cells (20,000 per well) were treated with C6-cHy-NPs (5 µL, 10x dilution), and the fluorescence of the samples was recorded after washing at different time intervals (0.25, 0.5, 1, 2, 3, 4, 5, 6, 8, 10, 24, and 48 h) of incubation. For microscopy analysis, the cells were seeded in a 6 well plate (1 × 10^5^ cells/well) and incubated overnight. The next day, the cells were washed and in each well media containing C6-cHy-NPs was added. At different time intervals (0.5, 1, 2, 3, 4, and 5 h), the cells in each well were washed with PBS and analyzed under the fluorescence microscope using a 20x lens (Revolve, Discover Echo Inc., San Diego, CA, USA) at λex 460 nm. Counterstain HOECHST-33342 (1 µg/mL, 10 min incubation in the dark, λex 350 nm) was used.

### Cell cytotoxicity study

HEI-OC1 cells were seeded in a 96-well plate (10,000 cells/well) and incubated overnight at 33 °C (5% CO_2_). The treatment groups were kept as untreated controls, *Cis*Pt only, *Cis*Pt + FL-cHy-NPs (20 µM equivalent to FL), *Cis*Pt + HK-NPs (20 µM equivalent to HK), *Cis*Pt + FL&HK-cHy-NPs (10 µM equivalent to FL&HK), and *Cis*Pt + STS (20 µM). First, 100 µL media containing 20 µM FL/HK/STS at the equivalent concentration of cHy-NPs or drugs were added to the respective wells. After 4 h of incubation, 100 µL media containing 50 µM *Cis*Pt was added. At the endpoint (48 h incubation), the cells were washed with fresh media two times and 100 µL media containing MTT reagent (0.5 mg/mL) was added to each well. After 4 h of incubation, the solubilization buffer (100 µL, DMSO: RIPA buffer; 50:50) was added to each well. After overnight incubation, the absorbance at 570 nm was recorded using UV-vis multi-well plate spectrophotometer (Synergy 2, BioTek, USA).

### Intracellular ROS generation, apoptosis, and necrosis

HEI-OC1 Cells (10,000 cells/well in 96-well plates) were treated with various treatment groups as described above in the cell cytotoxicity study section. The endpoints for ROS generation, apoptosis, and necrosis studies were set up at 24, 36, and 48 h, respectively. For ROS generation study, the cells were washed two times with fresh media and then culture media containing DCFH_2_-DA reagent (10 µM) was added to each well. After 30 min incubation in dark at 33 °C (5% CO2) the cells were washed with PBS three times. Fresh PBS (100 µL) was added and the fluorescence was recorded at λex/em 480/530 nm using a multi-well plate fluorescence spectrophotometer (Synergy 2, BioTek, USA). For apoptosis study, at the endpoint (24 h), the cells were washed three times and incubated with caspase 3/7 assay reagent according to the manufacturer’s protocol (#APT423, CaspaTag™ In Situ Caspase-3/7 Detection Kit, Sigma-Aldrich, Sant Louis, MO, USA) for 30 min [38]. After washing, the fluorescence was recorded at λex/em 480/530 nm using a multi-well plate fluorescence spectrophotometer (Synergy 2, BioTek, USA). To analyze the necrotic state of the cells, at the endpoint (48 h), the cells were washed using PBS and supplemented with fresh PBS containing PI (1 µg/mL). After 15 min of incubation in dark, the fluorescence was recorded at λex/em 530/615 nm using a multi-well plate fluorescence spectrophotometer (Synergy 2, BioTek, USA). The fluorescence microscopy analysis of ROS generation, apoptosis, and necrosis experiments was performed. Briefly, the nuclei of the cells from all the experiments were counterstained using HOECHST-33342 (1 µg/mL) for 15 min in the dark. The cells were observed under the fluorescence microscope using a 20X lens at the respective dye fluorescence filter as described above. For HOECHST-33342 λex/em 350/460 nm fluorescence filter was used.

### Western blot analysis

After the treatment endpoint, in each well, the cells were washed with PBS, and incubated with 100 µl mixture of 2X blue dye (90 µL, for recipe see SI section S3) and β-mercaptoethanol (10 µL) for 1 min at room temperature. The cells were collected using sterile cell scraper in microcentrifuge tube. The samples were centrifuged for 10 Sec (1000 rpm) and heated for 5 min at 100 °C. The samples were either loaded to the gel (SDS-PAGE) or stored at -20 °C for further use. After running the samples in precast SDS-PAGE gel (4–15% Mini-PROTEAN^®^ TGX™ Precast Protein Gels, BIO-RAD), the samples were transferred to the PVDF membrane using Trans-Blot^®^ Turbo™ Transfer System (BIO-RAD). The membranes were blocked using nonfat dried milk (5%) in TBS-T containing Tween 20 (0.1%) at room temperature for 1 h. After washing, the membranes were incubated with respective assay primary antibodies; anti caspase 3 (#14220s, 1:1000) and anti cleaved caspase 3 (#9664s, 1:1000),(all from Cell Signaling Technologies) diluted in nonfat dried milk (1.5%) in TBS-T, overnight at 4 °C. The membranes were then washed with TBS-T (4 times every 5 min) and incubated further with goat anti-rabbit (#12,004,161, BIO-RAD, 1:2500) or goat anti-mouse (#92,632,210, Li-Cor, 1:2500) secondary antibodies for 1 h. The membranes were washed using TBS-T (4 times every 5 min). The bands corresponding to the target expression were imaged by Biorad’s ChemiDoc Imaging System.

### Preparation of FL-cHy-NPs and HK-cHy-NPs embedded thermoresponsive hydrogel formulation

The thermoresponsive hydrogel i.e. thermogel was prepared using a previously described method with some modifications [39]. Briefly, poloxamer-407, poloxamer-188, and carbomer-940 were mixed at concentrations of 24, 15, and 0.1% w/v, respectively, in a cold (4 °C) colloidal solution of prepared cHy-NPs (FL-cHy-NPs and HK-cHy-NPs) and kept at 4 °C overnight. The thermoresponsive behavior of the prepared gels was characterized by maintaining them at 25 and 37 °C. The hydrogel formulations kept at 25 and 37 °C were instantly frozen using liquid nitrogen and then freeze-dried using a lyophilizer. The dried powder of the samples was spread on carbon tape, fixed on a mounting stub, and sputtered with gold [40]. The processed samples were then analyzed using high-resolution field emission scanning electron microscopy (FE-SEM; Neon 40EsB, Zeiss, Baden-Württemberg, Germany).

### Field emission scanning electron microscopy (FE-SEM) image based analytical study

Utilizing the high resolution field emission scanning electron microscopy images were analyzed for quantitative and qualitative comparisons. Images of the FL-cHy-NPs and HK-cHy-NPs hydrogels preparations were taken at a range of magnifications from 5 to 35 KX. Using the DigiM I2S software platform the 5KX magnification images, of the FL-cHy-NPs and HK-cHy-NPs hydrogels stored at 25 and 37 °C, were analyzed and segmented to determine the particle size and distribution throughout the visible scene. A combination of artificial intelligence (AI)-based image analytical techniques, including a deep learning semantic segmentation model, was used to determine the particle location within the scene. The deep learning model was trained using a supervised learning pattern, in which the inferred regions of the images were manually corrected and passed back to the model as additional training data. This process was repeated until a segmentation with little to no visible issues remained. After segmentation, morphology of the drug particles including their size, shape, and percentage was analyzed.

### In vitro drug release kinetics

Two 24 well plates were equipped with 6.5 mm inserts (Transwell^®^, Costar, Corning, NY, USA) having polycarbonate support membrane with 8 μm pores at the bottom (six in each plate). The developed hydrogel formulations containing FL-cHy-NPs and HK-cHy-NPs were filled into the inserts (*n* = 3, 100 µL each formulation in each insert). The insert served as the donor compartment while the well served as the receiver compartment. One plate was placed at 25 °C while the other one was kept at 37 °C. After 30 min, the receiver media (1 mL, PBS pH 7.4) was carefully filled from the side wall in each well (receiver compartment) containing the insert (donor compartment) filled with hydrogel formulation [40]. Other empty wells without inserts were filled with deionized water to maintain humidity in the plate which helped in avoiding the possible evaporation of water content from the hydrogel and receiver media. The samples (100 µL) were drawn from the receiver well at different time intervals (0.5, 1, 3, 6, 10, 24, 48, 72, 144, 312, 480, and 696 h) and stored at 4 °C. The sink condition of the receiver compartment was maintained by adding 100 µL of fresh PBS. The drug content (FL and HK) was extracted using dichloromethane (DCM; solvent-solvent extraction). After evaporating the DCM, the extracted FL and HK content was dissolved in methanol (100 µL, 100% LC-MS grade). The quantification of FL and HK in each sample was performed using HPLC and respective standard equation stated in the  HPLC method development section. The release kinetics of FL and HK from both hydrogels were calculated using the Korsmeyer-Peppas model (Eq. 7) [41].

6 $$\frac{{M}_{t}}{{M}_{\infty }}={k}_{1}{t}^{n}$$

where, ${M}_{t}$ is the mass of the drug released at time t; ${M}_{{\infty }}$ is the mass of the total drug; ${k}_{1}$ is the structural and geometric characteristic of the dosage form related constant; n is the release mechanism exponent.

### Otoprotection studies on zebrafish

*Z*ebrafish were maintained at Creighton University Animal Resource Facilities by standard methods and the performed experiments were in accordance with approved IACUC protocol by the Institutional Animal Care and Use Committee. Fish were maintained at 28.5 °C in E3 media with a 14-hour light/10-hour dark cycle [42].

Otoprotection studies were performed in 5–6 dpf wild-type (TuAB) fish as previously described [43]. Briefly, fish were pre-incubated for one hour in E3 media containing FL-cHy-NPs, HK-cHy-NPs, or FL&HK-cHy-NPs, and then co-incubated for 6 h with the corresponding cytoprotective molecules and clinical cisplatin 400µM. The amount of cHy-NPs taken as equivalent concentrations of FL and HK as follows: FL or HK = 33µM, 17µM, and 2µM; FL-+HK = 17 + 17µM, 8.5 + 8.5µM, and 3 + 3µM. Fish were also incubated with empty cHy-NPs as a control. After the treatment, fish were transferred to fresh E3 media for one hour to recover, followed by fixation (4% paraformaldehyde; PFA) and processing for confocal imaging. For the detection of neuromast hair cells, fish were immunostained for the hair cell marker, otoferlin (HCS-1, DSHB, 1:200 dilution). Hair cells were manually counted using a Zeiss AxioSkop 2 fluorescence microscope. The neuromasts inspected were part of the cranial system and included the otic, middle, and opercular neuromasts. Ten to twelve fish were used per treatment.

Confocal microscopy imaging was performed using a Zeiss LSM 700 confocal laser scanning microscope (CLSM). Images were captured at room temperature with automatically set sectioning and processed with ZEN black edition software. *Z*-stacks are presented as flat *Z*-projections. Final figures were assembled using Photoshop and Illustrator software (Adobe).

### Statistical analysis

GraphPadPrism 9 (San Diego, USA) was used for the statistical analysis of data through one-/two-way ANOVA. Data were presented as mean ± SD. The tests were validated using Šidák’s/Dunnet multiple comparison post-hoc test where *p* < 0.05 was considered to indicate a significant difference. The graphs and figures were drawn using GraphPad Prism 9, Origin Pro 9, Microsoft Office and Biorender.com.

## Results and discussion

### Analytical method development for flunarizine (FL) and honokiol (HK)

The HPLC-based analytical methods for qualitative and quantitative determination of FL and HK were successfully developed (Table S1). The HPLC chromatogram showed retention times of 1.72 and 2.22 min for FL and HK, respectively with a total acquisition time of 4 min (Figure S1). The standard equations for the quantitative determination of FL and HK in the samples were found to be ‘y = 34.897x + 1.553’ and ‘y = 33.84x + 1.5287’, respectively, where, y is the area of the curve and x is the concentration in µg/mL units (Figures S2 and S3). The LOD were 0.059 ± 0.013 and 0.038 ± 0.004 µg/mL for FL and HK, respectively while the LOQ were found to be as low as 0.179 ± 0.040 µg/mL (for FL) and 0.116 ± 0.012 µg/mL (for HK) (Tables S2 and S3). The developed analytical method was found to be highly sensitive and robust for the qualitative and quantitative characterization of FL and HK in various samples during the study.

### Preparation of FL-cHy-NPs and HK-cHy-NPs

**Fig. 1:**
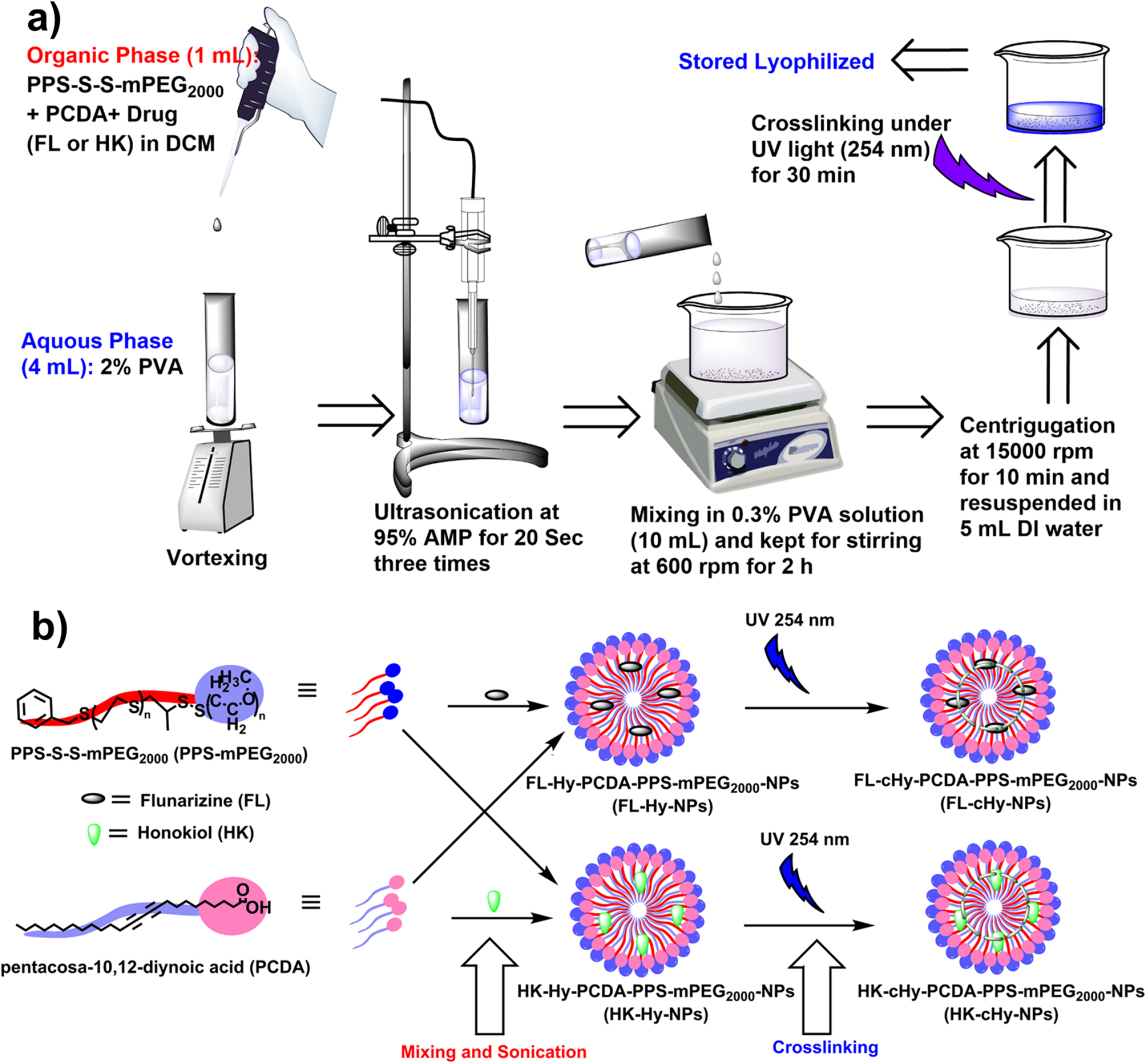
(**a**) Schematic representation for the synthesis of FL-cHy-NPs and HK-cHy-NPs. (**b**) Schematic of the arrangement of PCDA, PPS-mPEG_2000_, and encapsulated drugs (FL or HK) and subsequent crosslinking during the synthesis of FL-cHy-NPs and HK-cHy-NPs.

The FL-cHy-NPs and HK-cHy-NPs were successfully synthesized using the ultrasonic nanoprecipitation solvent evaporation method as depicted in Fig. 1a. During the synthesis of the cHy-NPs, the hydrophobic moieties of the PPS-mPEG_2000_ and PCDA molecules come together and form the core of the nanoparticles (NPs) while the hydrophilic moieties (PEG and -COOH) are arranged on the surface of the NPs in contact with the aqueous media. The FL and HK drugs are generally entrapped in the core of NPs during synthesis due to the hydrophobic nature of drugs. A change in color from pale white to dark blue was observed after UV irradiation (254 nm) for 30 min confirming the crosslinking of the PCDA molecules (Fig. 1b). The crosslinking of PCDA molecules stabilizes the NP preparation (cHy-NPs) in the solution and helps them to retain their morphological characteristics during storage and further processing (lyophilization, saline reconstitution, and entrapment in hydrogels, creams, capsules, etc.) of the formulation.

### DoE-CCD based formulation parameter optimization for the synthesis of FL-cHy-NPs and HK-cHy-NPs

**Fig. 2:**
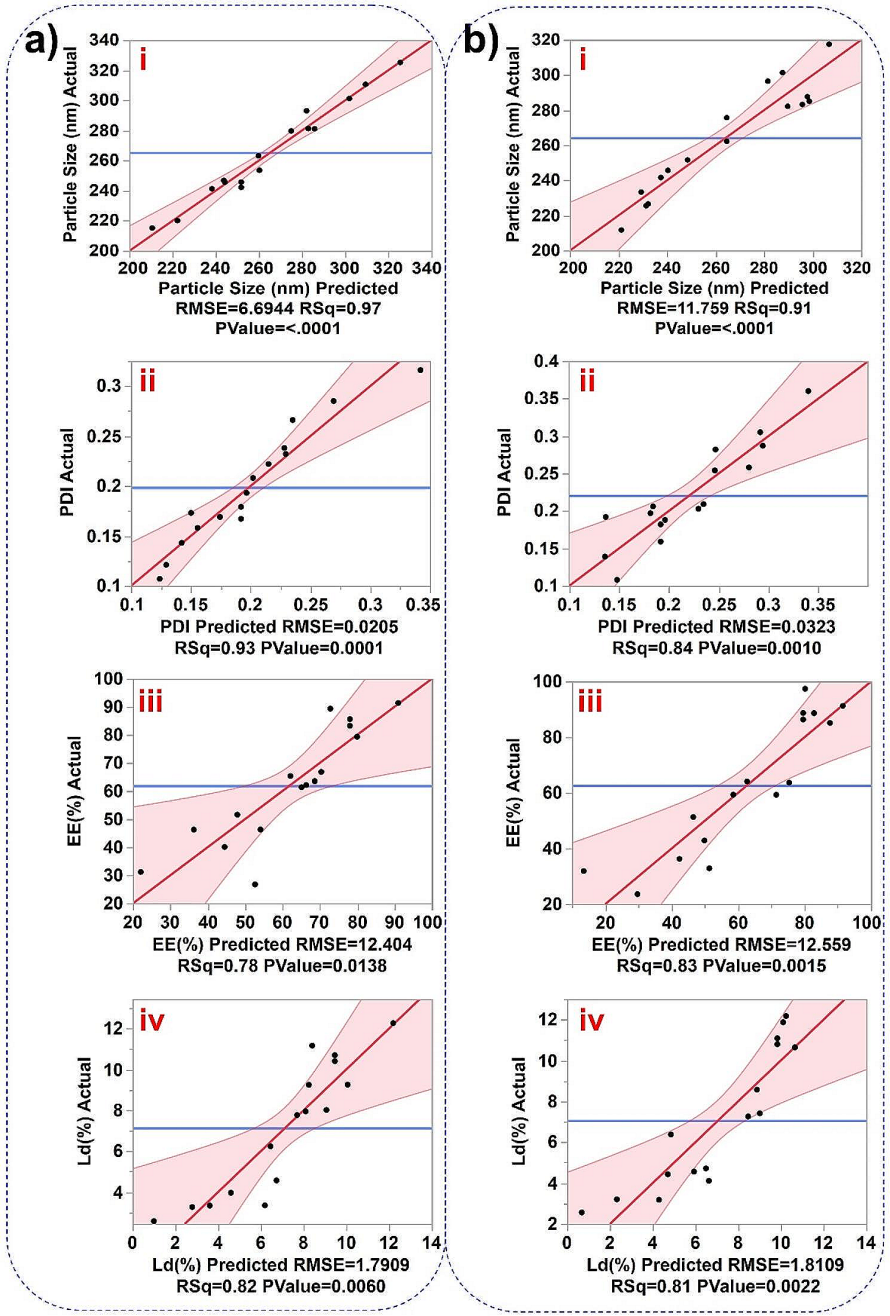
Actual vs. predicted plots of responses after performing all 16 experiments and running the Standard Least Square model with emphasis on effective screening. (**a**) Plots for FL-cHy-NPs. (**b**) Plots for HK-cHy-NPs. The responses for each NP preparation are presented as ‘i’ Particle Size (nm), ‘ii’ PDI, ‘iii’ EE (%), and ‘iv’ Ld (%). In each response plot, the black dots are showing the response values obtained after performing the experiments, the blue line is representing the null hypothesis and a red slanted line as alternative hypothesis. The fainted red area around the red line representing the 95% confidence region. Here, RMSE; Root Mean Squared Error showing the average measured values of difference between the predicted and actual values. RSq (R^2^) values under each response plot showing how close the data fitted to the regression line (alternative hypothesis).

Prior to the final synthesis of the cHy-NPs, the synthesis parameters were optimized using the design of experiment (DoE) approach. A central composite design (CCD) was employed for the optimization of the three factors i.e. concentration of PPS-mPEG_2000_, PCDA, and drugs (FL or HK) with respect to four responses (size, PDI, entrapment efficiency, and drug loading) for the synthesis of cHy-NPs. To optimize the synthesis of both FL-cHy-NPs and HK-cHy-NPs, the experiments (16 runs suggested by the software) with three different parameters namely PPS-mPEG_2000_ concentration, PCDA concentration (% w/w of PPS-mPEG_2000_) and drug concentration (FL or HK; % w/w of PPS-mPEG_2000_ and PCDA) were performed. The effects of various factors on the responses [particle size, PDI, encapsulation efficiency (EE%), and loading (Ld%)] were screened by running the Standard Least Square model. The actual vs. predicted value plots show both null hypothesis (response independent of factors, blue line) and alternative hypothesis (response dependent on factors, red line) in the graphs (Fig. 2). The 95% confidence region was also present in each plot above the alternative hypothesis (red) line. Visually, in each response plot, the null hypothesis line (blue line) was not contained within the red region (95% significance) suggesting that the test model was significant. This was confirmed by the Analysis of Variance (AVOVA), which suggested the *F*-test value for each plot was < 0.05 (Figure S14). Further, for a true fit, the lack of fit must be non-significant (*F* > 0.05), visually, the red line passess through the middle of the data points, and most of the response points present within the 95% confidence area near the red line. This observation suggested that the data was well fitting with the line and the lack of fit was not significant. This was confirmed by the Lack of Fit table of each response that showed *F* > 0.05 (Figure S15).

To further confirm the best fit of the model, Scatter Index (SIn) values for each response were calculated using Root Mean Squared Error (RMSE) and average values of each response. The SIn values of each response were ≤ 25% which confirmed that the CCD-based standard least square model could perform well for the factor optimization to obtain better responses in the NP development process (Table 1). Further, the analysis of residual vs. predicted plots showed the random distribution of the response values around the residual line (0-line) which suggested a good fit model for the respective response (Figure S16). Moreover, the studentized residual plot for each run (row) did not show significant outliers from the 95% simultaneous limits estimated by the Bonferroni test (red lines in the plot) (Figure S17).

**Table 1:** The average values of the responses, Root Mean Square Error (RMSE), and Scatter Index (SIn)

S.No.	Response	Mean response values of all 16 runs		RMSE		SIn	
		FL-cHy-NPs	HK-cHy-NPs	FL-cHy-NPs	HK-cHy-NPs	FL-cHy-NPs	HK-cHy-NPs
**1**	Particle Size (nm)	265	264	6.69	11.76	2.5%	4.4%
**2**	PDI	0.199	0.221	0.02	0.032	10%	15%
**3**	EE (%),	61.9	62.68	12.4	12.56	20%	20%
**4**	Ld (%)	7.13	7.06	1.79	1.81	25%	25%

**Fig. 3:**
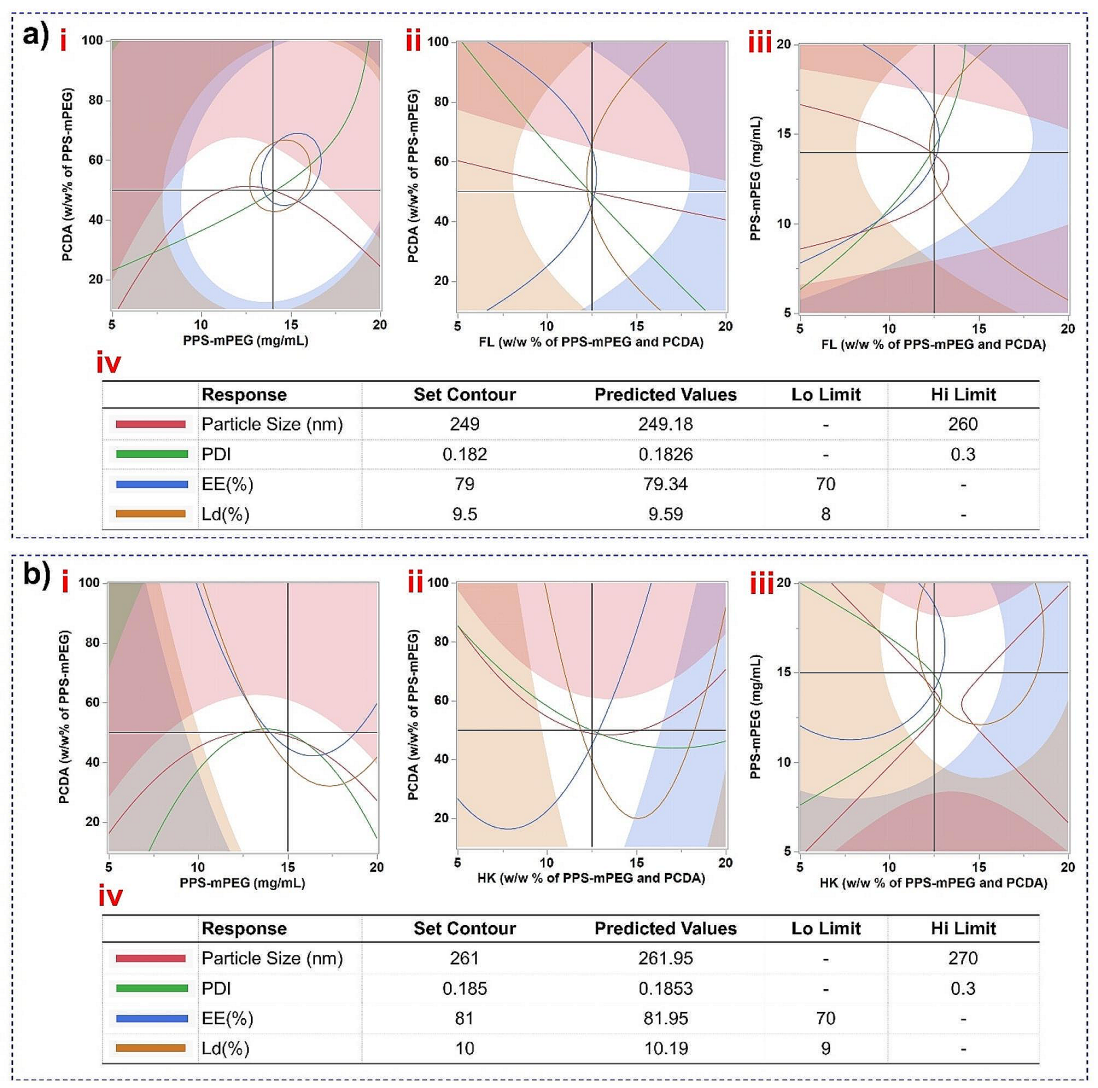
Contour profiler graphs showing effect of various factors on the responses during the synthesis of (**a**) FL-cHy-NPs, and (**b**) HK-cHy-NPs. In each figure contour graphs showing responses against factors ‘i’ PCDA vs. PPS-mPEG_2000_, ‘ii’ PCDA vs. Drug (FL or HK), and ‘iii’ PPS-mPEG_2000_ vs. Drug (FL or HK) are shown. Table ‘iv’ shows the set values, predicted values, low (Lo) limit and high (Hi) limit of each response fixed during the preparation of the plots. The shaded portions of each plots show the responses beyond the fixed Lo or Hi limits. The horizontal and vertical black lines showing the optimized conditions of the respective factor where they are crossing ‘y’ and ‘x’ axis, respectively

**Fig. 4:**
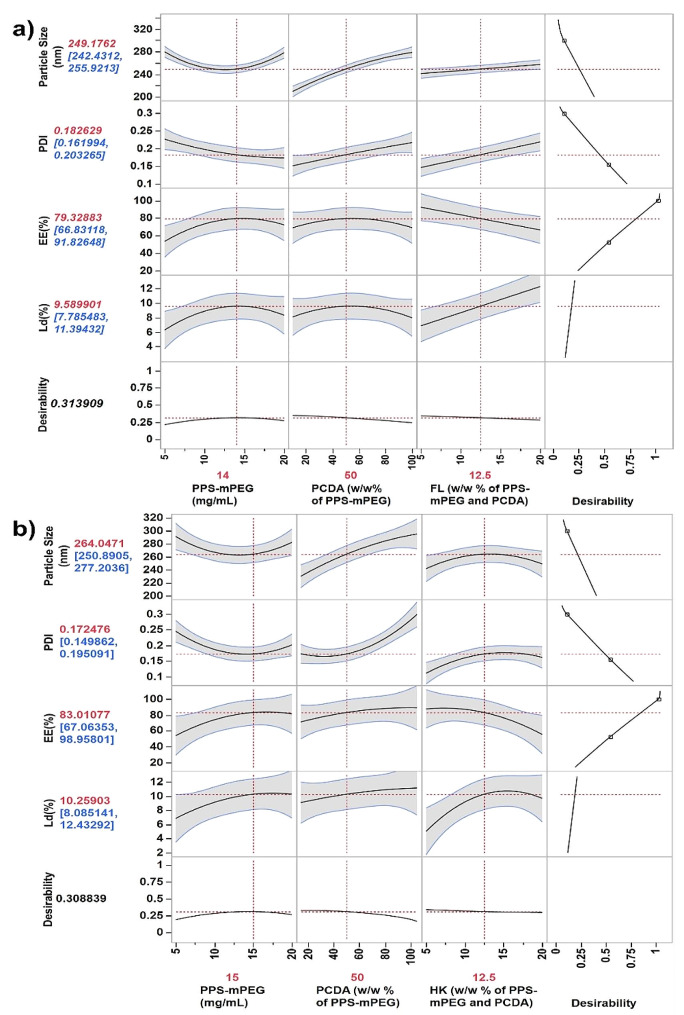
Prediction profiler graphs showing optimized conditions calculated by the quadradic model using the central composite design (CCD) by JMP^®^ pro software. The values denoted by red color showing the most optimal condition and predicted results (responses: particle size, PDI, encapsulation efficiency (EE%), and drug loading (Ld%), while the value denoted by blue color shows the range of the response values at the same synthesis conditions. The graph shows conditions for optimal synthesis of  (a) FL-cHy-NPs (**b**) HK-cHy-NPs.

After ensuring the good fit model, the response contour graphs were plotted to determine the effect of various factors on the responses [particle size, PDI, encapsulation efficiency (EE%), and loading (Ld%)]. It was observed that by setting the contour near the predicted values for the highest desirable response, contour graphs for mixtures of all the responses were plotted. Using these plots, the highly favorable factors [(polymers; PCDA, PPS-mPEG_2000_, and drugs; FL and HK)] concentrations were obtained (Fig. 3). Furthermore, the optimal factor conditions were confirmed using a prediction profiler graphical tool **(**Fig. 4**)**. The optimized concentrations of PPS-mPEG_2000_, PCDA, and FL were 14 mg/mL, 50% (w/w of PPS-mPEG_2000_) and 12.5% (w/w of PPS-mPEG_2000_ and PCDA), respectively, for the synthesis of FL-cHy-NPs with ~ 250 nm size, 0.18 PDI, 79% EE and 9.6% Ld (Fig. 4a). Similarly, to synthesize HK-cHy-NPs with ~ 260 nm size, 0.18 PDI, 82% EE and 10% Ld, the concentrations of PPs-mPEG_2000_, PCDA, and HK, 15 mg/mL, 50% (w/w of PPS-mPEG_2000_) and 12.5% (w/w of PPS-mPEG_2000_ and PCDA), respectively, were found to be optimal (Fig. 4b). The effect of factors on the responses was observed. irrespective of FL or HK drugs, almost all of the factors affected the responses similarly during the synthesis of both FL- and HK-loaded cHy-NPs (Fig. 4a and b). Increasing the PPS-mPEG_200__0_ concentration elicited decreased size and PDI values up to 15 mg/mL, and increased encapsulation and loading efficiencies. After that the size and PDI increased and, encapsulation and loading decreased. Thus, 15 mg/mL PPS-mPEG_2000_ concentration was considered optimal for NPs synthesis. Increasing PCDA concentration, the size and PDI values increased, however, the EE and Ld were also increased up to 50% w/w, then decreased (during FL-cHy-NPs preparation) and no significant change was observed during preparation of HK-cHy-NPs. Therefore 50% w/v concentration of PCDA was considered optimal for the preparation of both FL-cHy-NPs and HK-cHy-NPs. During the synthesis of FL-cHy-NPs, increasing in concentration of FL elicited increased size and PDI values. It was also responsible for increased Ld (%) value, however, (EE%) was decreasing. Therefore to keep desired Size, PDI, EE(%), and Ld(%) values, the FL concentration was set at 12.5% (w/w ) of polymers (both PPS-mPEG_2000_ and PCDA). During optimization of HK concentration, it was observed that increasing HK concentration prompted increased size, PDI, and Ld(%) up to 12.5% w/w of polymers after that decrease in these values observed. There was a nonsignificant decrease in EE(%) observed till 12.5% w/w of HK, however, after that significant decrease in EE(%) was observed. These results suggested the optimal concentration of HK was 12.5% w/w of both PPS-mPEG_2000_ and PCDA polymers. The initial increase in PPS-mPEG_2000_ concentration led to the reduction in size and PDI might be due to the ease and compact self-assembly with respective PCDA concentration. The amphiphilic nature of PPS-mPEG_2000_ is comparatively more than PCDA and it is speculated that an increase in the respective concentration of PPS-mPEG_2000_ could be responsible for easy self-assembly of NPs. Further increase in the concentration might be responsible for the increased size of the NPs because of the formation of bulkier NPs or less dense structural arrangement. The decrease in encapsulation with the increased polymeric concentration confirmed that there were some structural deformities occurred with the increased concentration of polymers that were responsible for the leaching of added drug (FL or HK) molecules.

### Characterization of FL-cHy-NPs and HK-cHy-NPs

**Fig. 5:**
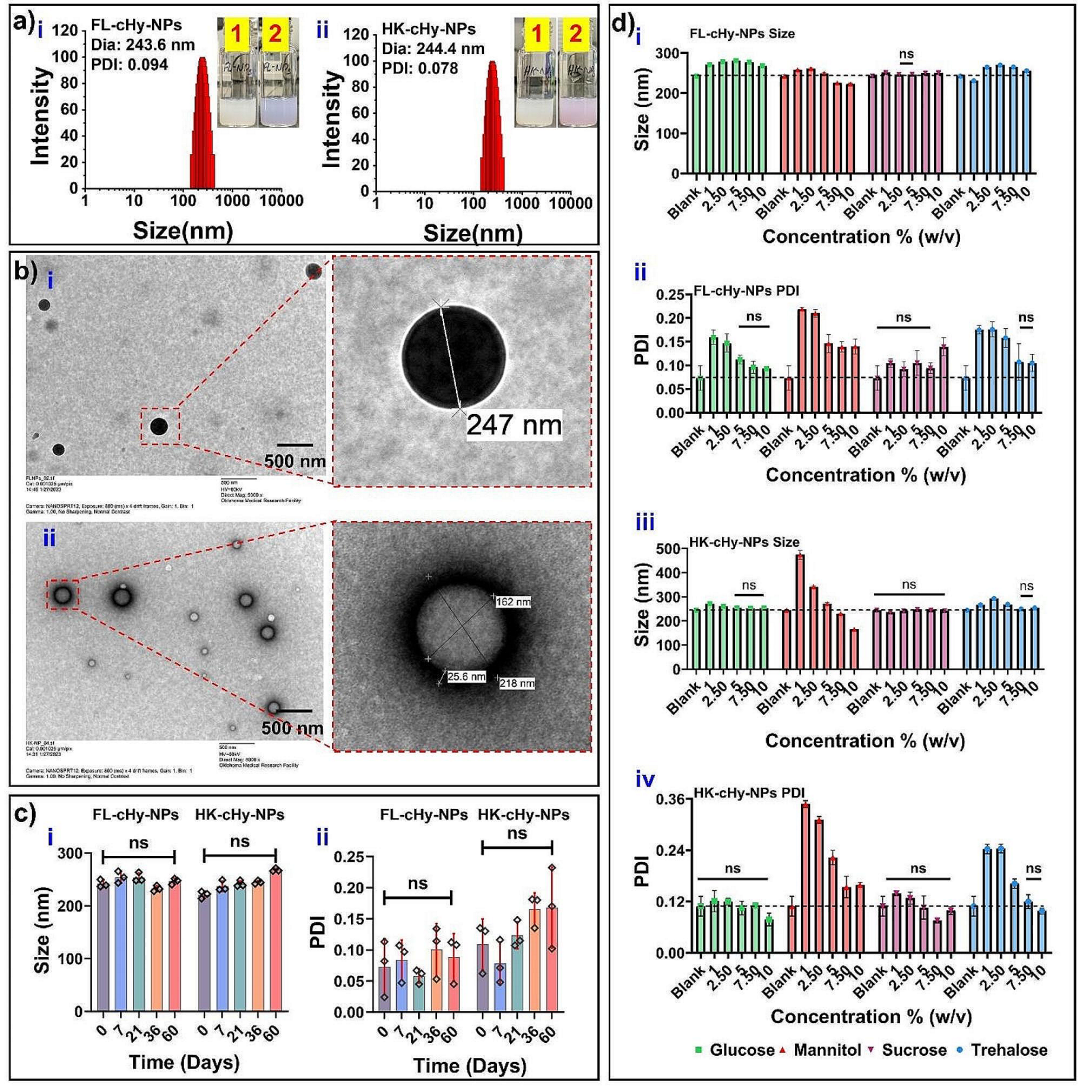
(**a**) Size distribution graphs of (i) FL-cHy-NPs and (ii) HK-cHy-NPs showing the average diameter of the NPs and polydispersity index (PDI). The inset figure shows the visual appearance of the colloidal solution of the same cHy-NPs before (1) and after (2) crosslinking. (**b**) Figures showing TEM images of developed cHy-NPs confirm their spherical shape and intact integrity (i) TEM Image of the FL-cHy-NPs (scale 500 nm) and inset; enlarged NP. (ii) TEM image of the HK-cHy-NPs (scale 500 nm) and inset; enlarged NP. (**c**) The stability of synthesized FL-cHy-NPs and HK-cHy-NPs in the colloidal solution stored at 4° C: (i) the average size and (ii) PDI values of the cHy-NPs at 0, 7, 21, 36 and 60 days of storage. The means of each sample were compared using one-way ANOVA and using Dunnett multiple comparison post hoc test with a family-wise alpha threshold confidence level of 0.05 (95% confidence interval). Here, all the values are showing no difference (ns; *p* > 0.05) with each other suggesting the stability of the cHy-NPs at different time points. (**d**) Effect of various cryoprotectants on the size and PDI of the NP formulations after lyophilization. The graphs are showing effect of cryoprotectants on the (i) size of FL-cHy-NPs, (ii) PDI values of FL-cHy-NPs, (iii) size of HK-cHy-NPs, and (iv) PDI value of HK-cHy-NPs. The data were analyzed using two-way ANOVA and the group sample means were compared with the blank sample (non-lyophilized) by Dunnett’s multiple comparison post-hoc test. The family-wise alpha threshold confidence level was adjusted to 0.05 (95% confidence interval) during the analysis. Here, all the values are showing no difference (*p =* ns > 0.05) with each other suggesting the optimal concentration of the respective cryoprotectant to stabilize the NP preparation (size and PDI).

After preparation, the FL-cHy-NPs and HK-cHy-NPs were carefully characterized. The hydrodynamic diameters of the FL-cHy-NPs and HK-cHy-NPs determined using DLS were 243.6 ± 1.9 and 244.4 ± 2.5 nm, respectively. The polydispersity index (PDI) values of the FL-cHy-NPs and HK-cHy-NPs were found to be 0.094 ± 0.055 and 0.078 ± 0.045, respectively (Fig. 5a). These PDI values (≤ 0.3) suggest that the synthesized cHy-NPs were highly dispersed in the colloidal solution and had a uniform size distribution. The TEM analysis of both FL-cHy-NPs and HK-cHy-NPs confirmed the spherical morphology after the final preparation of the cHy-NPs (Fig. 5b). The encapsulation efficiencies (EE%) of FL and HK in the respective cHy-NPs were found to be 78.3 ± 8.1 and 80.52 ± 8.4%, respectively. The drug loading (Ld%) of FL and HK in the respective cHy-NPs was 9.47 ± 1.21 and 9.73 ± 1.41%, respectively. The size, PDI, encapsulation efficiency and loading values of the synthesized cHy-NPs were shown similar to the predicted values of prediction profiler. These results confirmed that the validity of the obtained predicted factor values by CCD were feasible to synthesize FL-cHy-NPs and HK-cHy-NPs. Further, the cHy-NPs were stable in the colloidal solution for more than 60 days at 4 °C (Fig. 5c).

### Stability and freeze-drying studies

Although the prepared FL-cHy-NPs and HK-cHy-NPs were highly stable in the solution, their long-term storage was possible after lyophilization, because, in the solution, the release of the drugs from the FL-cHy-NPs and HK-cHy-NPs could not be controlled. Therefore, lyophilization study of the prepared FL-cHy-NPs and HK-cHy-NPs was performed using different cryoprotectants (glucose, mannitol, sucrose, and trehalose) at different concentrations (1, 2.5, 5, 7.5, and 10% w/v). After the reconstitution of the cHy-NPs, the results suggested that sucrose and glucose as cryoprotectants, had relative stabilization potential. Nevertheless, it did not have a significant protective effect at lower concentrations (< 5% w/v). Other cryoprotectants (mannitol and trehalose) were not able to maintain the appropriate size and PDI for the lyophilization of FL-cHy-NPs and HK-cHy-NPs. Therefore, sucrose at 2.5 and 5% w/v had a cryoprotective effect on the synthesized FL-cHy-NPs and HK-cHy-NPs (Fig. 5d).

### Cellular internalization study

**Fig. 6:**
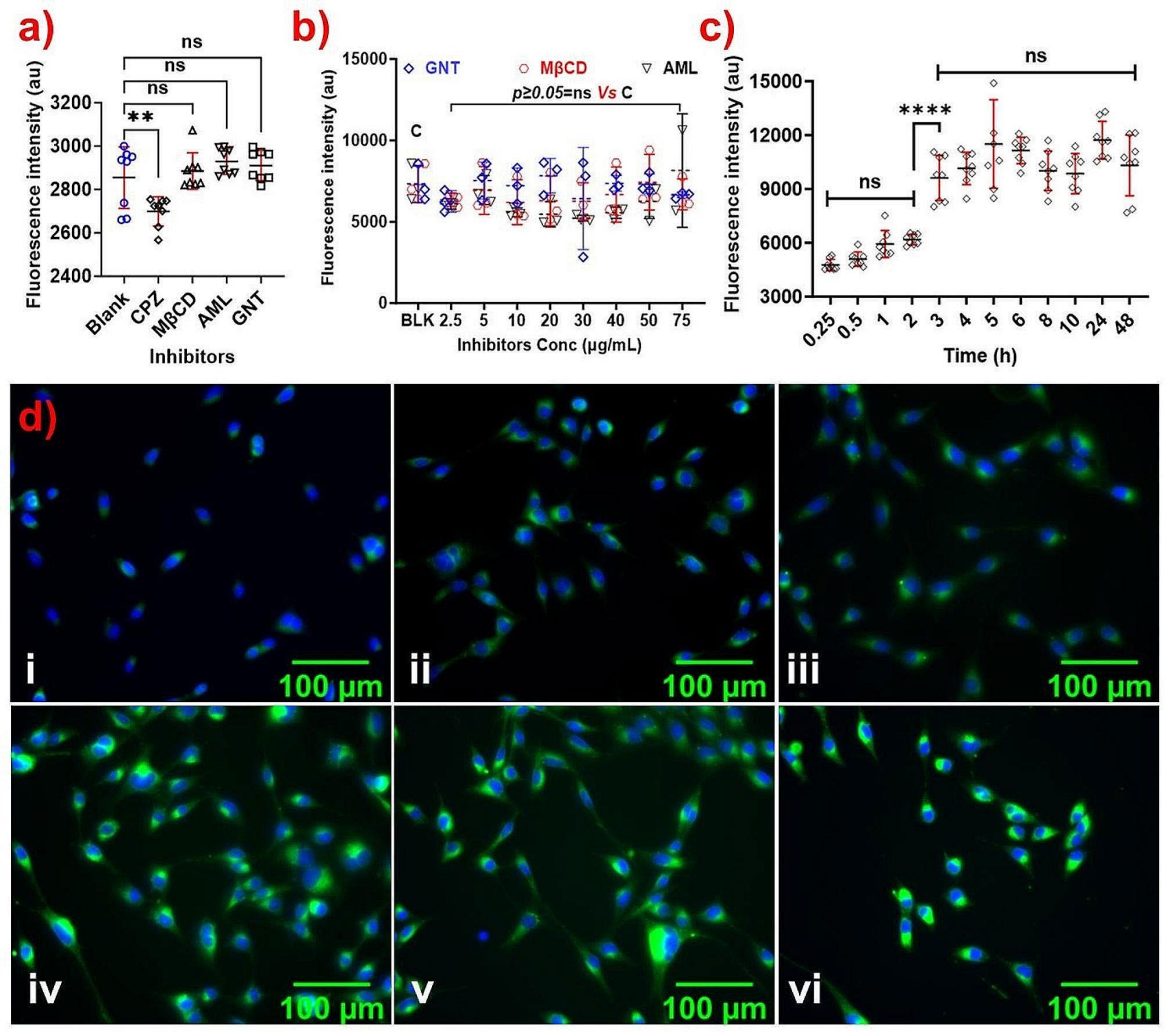
Cellular internalization of the synthesized cHy-NPs. (**a**) The fluorescence intensity graph of cellular internalization of C6-cHy-NPs in presence of various pathway inhibitors (CPZ, MβCD, AML, and GNT) in comparison with untreated cells. The CPZ showed significant inhibition of fluorescence intensity whereas, no significant difference in fluorescence intensities is showing up in MβCD, AML, and GNT treated groups. (**b**) The graph shows effect of various concentrations of pathway inhibitors on cellular internalization of NPs. No significant effect of MβCD, AML, and GNT was shown at the concentration from 2.5–75 µg/mL. (**c**) The graph shows time-dependent cellular internalization of C6-cHy-NPs at different time intervals (0.25, 1, 2, 3, 4, 5, 6, 8, 10, 24, and 48 h). (**d**) The fluorescence microscopy images of HEI-OC1 cells (scale 100 μm) after incubation with C6-cHy-NPs at different time intervals (i) 0.5 h, (ii) 1 h, (iii) 2 h, (iv) 3 h, (v) 4 h and (vi) 5 h); (green fluorescence, using FITC filter). The blue stain is showing the nuclei of the cells stained by HOECHST-33342 (using DAPI filter). [Data in the graphs were compared using two-way ANOVA and each group was compared with the ‘blank’ or control group “C” by applying Šidák multiple comparisons post-hoc tests. The family-wise alpha threshold confidence level was adjusted to 0.05 (95% confidence interval) during the analysis. (Here *p* ≥ 0.05 = ns, not significant)].

The cellular internalization of developed NPs plays a crucial role in drug delivery in the cell cytoplasm and directly affects their pharmacological actions. The main pathways associated with the cellular internalization of various NPs are Clathrin-mediated endocytosis, caveolin/cholesterol-dependent endocytosis, Macropinocytosis, and Caveolin mediated endocytosis, which can be inhibited by Chlorpromazine (CPZ), Methyl β Cyclodextrin (MβCD), Amiloride (AML), and Genistein (GNT), respectively [44]. To study the major pathway associated with cHy-NPs cell internalization, fluorescent-labeled (Coumarin-6; C6-loaded) cHy-NPs were incubated with or without pathway inhibitors. In the fluorescence spectroscopy analysis of the HEI-OC1 cells that were pretreated with MβCD, AML, and GNT, no significant change in the cellular internalization of C6-cHy-NPs was observed. However, in the CPZ-pretreated HEI-OC1 cells, significantly reduced cellular internalization of C6-cHy-NPs was observed (Fig. 6a). Therefore, it may be concluded that the pathway accompanying the cellular internalization of FL-cHy-NPs and HK-cHy-NPs was Clathrin-mediated endocytosis. These results corroborate the findings of other PEGylated NP preparations suggesting that the main cellular internalization pathway of the PEGylate NP preparations is Clathrin-mediated endocytosis [45]. To confirm the specific participation of the clathrin-mediated pathway and rule out the involvement of any other pathway, the study was repeated with various pathway inhibitors (MβCD, AML, and GNT) at different concentrations (2.5 to 75 µg/mL). There was no significant change in the cellular internalization of C6-cHy-NPs observed compared to inhibitor untreated cells even at higher inhibitor concentrations (Fig. 6b). Therefore, it was confirmed that Clathrin-mediated endocytosis was the main internalization pathway for cHy-NPs. Further, the optimal time for the cellular internalization of cHy-NPs was determined by recording the fluorescence emission at different time intervals. A notable increase in fluorescence intensity was observed up to 3 h of incubation after that there was no significant change noticed. Furthermore, the lack of the critical changes in fluorescence intensity after 48 h confirmed the retention of the cHy-NPs in the cells for a longer time (Fig. 6c). These results suggest that the efficient cellular internalization of cHy-NPs in the HEI-OC1 cells can be achieved within 3 h of incubation. These findings were further confirmed using fluorescence microscopy analysis of the HEI-OC1 cells. Similar to fluorescence spectroscopy, a significant increase in fluorescence was observed until 3 h of incubation and after which no significant change in the fluorescence intensity in the cells was observed in the microscopy images (Fig. 6d). Moreover, the results of fluorescence microscopy and spectroscopy suggest that the cHy-NPs did not expel out from the cells over time as no significant change in the fluorescence was observed until 5 h of incubation. Overall, these results demonstrated that the developed cHy-NPs could efficiently deliver the loaded drugs (FL and HK) to inner ear hair cells within 3–6 h of administration.

### Cisplatin-induced cytotoxicity protection

**Fig. 7:**
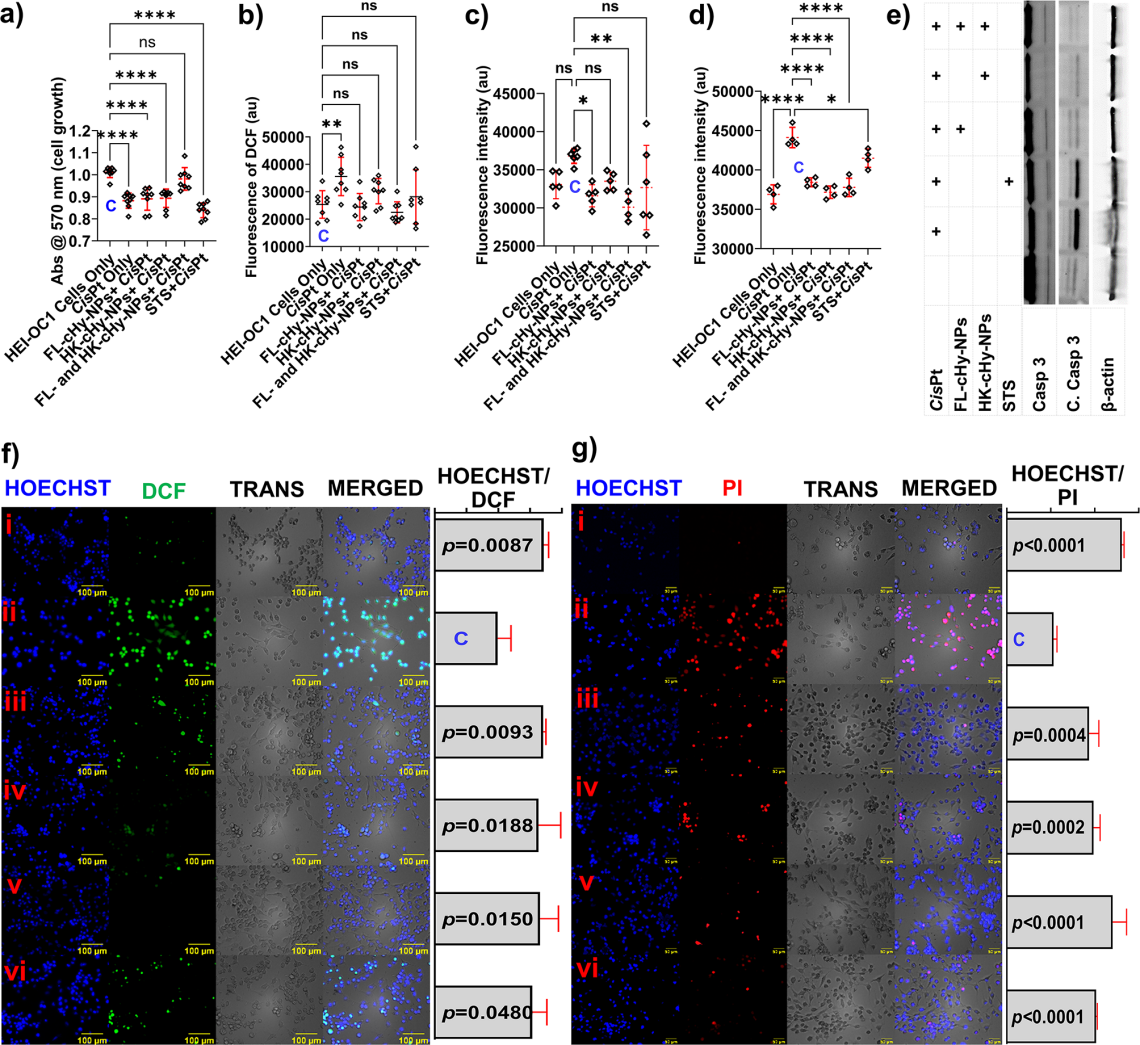
(**a**) Cell growth of various treatment groups determined using MTT assay. The treatment group of those treated with the combination of FL-cHy-NPs and HK-cHy-NPs did not show a significant reduction in cell growth compared to the control (*p* > 0.05, ns). Also, the combined formulation protects the cells from both 50 and 100 µM concentrations of *Cis*Pt. Therefore, the treatment of cells with the combination of FL-cHy-NPs and HK-cHy-NPs has significant protection efficacy against *Cis*Pt-induced cell death compared to FL-cHy-NPs or HK-cHy-NPs alone. (**b**) Intracellular ROS generation assay showing significant ROS generation in the *Cis*Pt only treated cells. Significantly low ROS generation was shown in the cells that were treated with FL-cHy-NPs, HK-cHy-NPs, and STS. (**c**) The graph showing the fluorescence intensity of MitoSOX reagent corresponded to mitochondrial superoxide generation. The assay suggested significantly low superoxide generation in FL-cHy-NPs and FL/HK-cHy-NPs treatment groups. (**d**) The graph showing caspase 3/7 activation in different treatment groups. The caspase 3/7 activation was significantly lowered in the cells that were treated with FL-cHy-NPs, HK-cHy-NPs, and STS. The data were compared using two-way ANOVA and each group was compared with the control group “C” by applying Dunnett’s multiple comparisons post-hoc tests. The family-wise alpha threshold confidence level was adjusted to 0.05 (95% confidence interval) during the analysis. (For 'a-d' data presented as Mean ± SD; Asterisk; ****, *p* < 0.0001; **, *p* < 0.005; *, *p* < 0.05; ns=not significant). (**e**) The western blot analysis of the samples after respective treatment with FL-cHy-NPs and HK-cHy-NPs alone or in combination. The blots are showing proteins associated with apoptotic caspase-3 pathway and control β-actin. (**f**) The fluorescence microscopy images (scale bar 100 μm) are showing the effect of developed FL-cHy-NPs and HK-cHy-NPs individually, and in combination on the *Cis*Pt-induced generation of ROS in HEI-OC1 cells. The first column is showing the cell nuclei stained with HOECHST-33342. The second column is showing the fluorescence of DCF (a ROS marker) in the cells. The third column is showing the cells under transmittance light. The fourth column is showing the overlay of columns 1, 2, and 3. The last column is showing a graph of the ratio of normalized intensities of overall cells and the cells that were producing ROS (getting stained with DCFH-DA). (i) Blank untreated cells: the cells that were not treated with any of the NP preparation or *Cis*Pt did not show significant ROS generation. The ratio of normalized intensities of total cells and DCF-stained cells was 1.71 ± 0.08. (ii) *Cis*Pt treated cells: The cells that were treated with *Cis*Pt only showed significant ROS generation. The ratio of normalized intensities of total cells and DCF-stained cells was 0.99 ± 0.21. (iii) FL-cHy-NPs and *Cis*Pt treated cells: the cells treated with FL-cHy-NPs and *Cis*Pt did not show significant ROS generation. The ratio of normalized intensities of total cells and DCF-stained cells was 1.71 ± 0.04. (iv) HK-cHy-NPs and *Cis*Pt treated cells: the cells treated with HK-cHy-NPs and *Cis*Pt did not show significant ROS generation. The ratio of normalized intensities of total cells and DCF-stained cells was 1.63 ± 0.35. (v) FL-cHy-NPs, HK-cHy-NPs and *Cis*Pt treated cells: the cells treated with FL-cHy-NPs, HK-cHy-NPs and *Cis*Pt did not show significant ROS generation. The ratio of normalized intensities of total cells and DCF-stained cells was 1.66 ± 0.29. (vi) STS and *Cis*Pt treated cells: the cells treated with STS and *Cis*Pt did not show significant ROS generation. The ratio of normalized intensities of total cells and DCF-stained cells was 1.53 ± 0.23. (**g**) The fluorescence microscopy images (scale bar 50 μm) showed the effect of developed FL-cHy-NPs and HK-cHy-NPs individually and in combination on the *Cis*Pt-induced HEI-OC1 cells cytotoxicity. The first column is showing the cell nuclei stained with HOECHST-33342. The second column is showing the fluorescence of PI (a dead cell marker) in the cells. The third column is showing the cells under transmittance light. The fourth column is showing the overlay of columns 1, 2, and 3. The last column is showing a graph of the ratio of normalized intensities of overall cells and the cells that were dead (getting PI stain). (i) Blank untreated cells: the cells that were not treated with any of the NP preparations or *Cis*Pt did not show significant cell death. The ratio of normalized intensities of total cells and PI-stained cells was 2.62 ± 0.05. (ii) *Cis*Pt treated cells: The cells that were treated with *Cis*Pt only showed significant cell death. The ratio of normalized intensities of total cells and PI-stained cells was 1.06 ± 0.08. (iii) FL-cHy-NPs and *Cis*Pt treated cells: the cells treated with FL-cHy-NPs and *Cis*Pt did not show significant cell death. The ratio of normalized intensities of total cells and PI-stained cells was 1.87 ± 0.22. (iv) HK-cHy-NPs and *Cis*Pt treated cells: the cells treated with HK-cHy-NPs and *Cis*Pt did not show significant cell death. The ratio of normalized intensities of total cells and PI-stained cells was 1.97 ± 0.15. (v) FL-cHy-NPs, HK-cHy-NPs and *Cis*Pt treated cells: the cells treated with FL-cHy-NPs, HK-cHy-NPs and *Cis*Pt did not show significant cell death. The ratio of normalized intensities of total cells and PI-stained cells was 2.41 ± 0.0.32. (vi) STS and *Cis*Pt treated cells: the cells treated with STS and *Cis*Pt did not show significant cell death. The ratio of normalized intensities of total cells and PI-stained cells was 2.03 ± 0.02. [The microscopy data were compared using one-way ANOVA and each group was compared with the control group (c; *Cis*Pt only treated) by applying Dunnett’s multiple comparisons posthoc test. The family-wise alpha threshold confidence level was adjusted to 0.05 (95% confidence interval) during the analysis and *p* < 0.05 was considered as a significantly different group].

Cisplatin (*Cis*Pt) induces cell apoptosis and necrosis in the inner ear hair cells leading to permanent hearing loss [46, 47]. We investigated the cytoprotective effect of FL-cHy-NPs and HK-cHy-NPs against *Cis*Pt-induced cytotoxicity on HEI-OC1 cells. The results suggested that the combination of FL-cHy-NPs and HK-cHy-NPs showed a significant cytoprotective effect compared to FL-cHy-NPs and HK-cHy-NPs alone. It was also observed that the cytoprotective effect of FL-cHy-NPs and HK-cHy-NPs against *Cis*Pt-induced cytotoxicity was even higher than the recently approved treatment regimen sodium thiosulfate (STS) [5, 48] (Fig. 7a). The considerable cytoprotective effect might be achieved due to the ROS detoxification effect related to the cellular deacetylase (sirtuin 3; Sirt-3) activation in the presence of HK  [19] . Moreover, a combined effect associated with the effect of FL was expectedly played a substantial role in cytoprotecting via activation of heme oxygenase – 1 (HO-1) through Nrf2 mediated transcription [17]. Overall, enhanced cytoprotecting action against *Cis*Pt-induced cytotoxicity in HEI-OC1 (inner ear hair cells) can be achieved by co-delivering FL-cHy-NPs and HK-cHy-NPs.

### Inhibitory effects of intracellular ROS generation, apoptosis and necrosis

The high concentration of ROS in the cells may lead to creating oxidative stress in the cells. Therefore, we investigated the generation of intracellular ROS in different treatment groups using the DCFH_2_-DA assay method. After 24 h of incubation (post *Cis*Pt addition), the untreated HEI-OC1 cells showed significantly lowered ROS generation compared to *Cis*Pt only-treated cells. The treatment groups with FL-cHy-NPs and HK-cHy-NPs (alone or in combination) and STS did not show a significant ROS generation compared to *Cis*Pt-only treated cells (Fig. 7b and f). Furthermore, the MitoSOX assay was employed to confirm the quenching effect of ROS generation. The result of the study suggested that the cells those were treated with FL-cHy-NPs, HK-cHy-NPs and STS showed significantly reduced mitochondrial superoxide compared to *Cis*Pt-only treated cells (Fig. 7c). After determining the effect of FL-cHy-NPs, HK-cHy-NPs and STS on *Cis*Pt-induced ROS generation in the cells, their significance on cell cycle and survival was further investigated. The cellular apoptosis study was done using a caspase 3/7 assay. The activation of caspase 3/7 was considered the confirmatory marker of the apoptotic stage of the cells (the higher the activation the higher the apoptosis). The results of the caspase-3/7 assay suggested that the cells that were treated with the FL-cHy-NPs and HK-cHy-NPs (alone or in combination) and STS showed significantly reduced signals of caspase 3/7 activation compared to the *Cis*Pt only treated cells (Fig. 7d). Moreover, significant reduction in cleaved caspase 3 protiens was observed in the FL-cHy-NPs and HK-cHy-NPs treatment groups, alone or in combination. Interestingly, a significant reduction of cleaved caspase-3 was observed in the combination treatment as compared to the groups that were treated alone. These results, in corroboration with cell cytotoxicity and apoptosis study data, confirm that the combination of FL-cHy-NPs and HK-cHy-NPs provides better protection against *Cis*Pt induced toxicity (Fig. 7e). Therefore, it may be concluded that the developed FL-cHy-NPs and HK-cHy-NPs were efficiently able to protect the HEI-OC1 cells from *Cis*Pt-induced cytotoxicity by quenching ROS generation and the subsequent reduction of oxidative stress which were responsible for the activation of apoptotic pathways. Finally, the cytoprotective effect of the developed FL-cHy-NPs and HK-cHy-NPs was investigated by determining cell necrosis. The PI staining assay was done to confirm the cytoprotective effect of developed FL-cHy-NPs, HK-cHy-NPs after the treatment with FL-cHy-NPs, HK-cHy-NPs, STS, and *Cis*Pt. The cells were stained with HOECHST-33342 and PI stains after 48 h of treatment. Compared to the untreated cells, a significant necrotic cell population was observed in the group treated with the *Cis*Pt only. However, cell groups that were treated with FL-cHy-NPs and HK-cHy-NPs (alone or in combination ) and STS (Fig. 7g) prior to the *Cis*Pt exposure, did not show significant necrotic cell population. Therefore, our investigation confirmed that the cells treated with FL-cHy-NPs and HK-cHy-NPs (alone or in combination) showed a significant cytoprotective effect against *Cis*Pt-induced cytotoxicity that was comparable with the approved STS treatment.

### FL- and HK-cHy-NPs embedded thermoresponsive hydrogel formulation

**Fig. 8:**
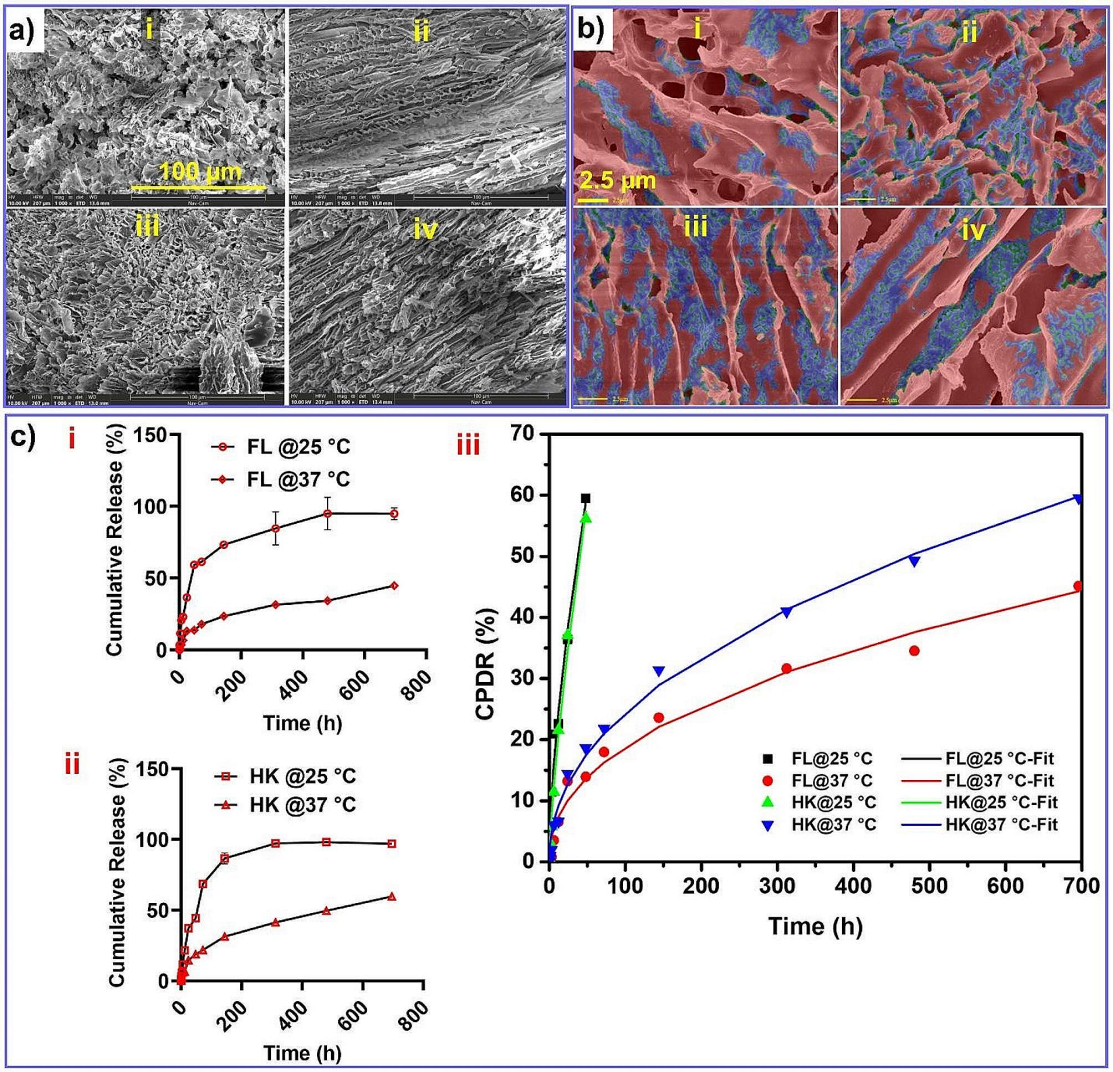
**(a)** SEM analysis of hydrogel after lyophilization (scale 100 μm); SEM images of FL-cHy-NPs kept at (i) 25 °C, and (ii) 37 °C; SEM images of HK-cHy-NPs kept at (iii) 25 °C, and (iv) 37°C. **(b)** FE-SEM images analysis of cHy-NPs embedded hydrogel formulation by deep learning segmentation (scale 2.5 μm); Segmentation of FL-cHy-NPs at (i) 25 °C, and (ii) 37 °C; Segmentation of HK-cHy-NPs at (iii) 25 °C, and (iv) 37 °C. In each of these segmentations blue regions represent deep learning segmentation model identified as particles. **(c)** In vitro drug release study of (i) FL, and (ii) HK at 25 °C and 37 °C, respectivelyfor up to 696 h (29 days). (iii) The graph showing fitting of cumulative percent drug release (CPDR) of FL and HK release with the Korsmeyer-Peppas Model form the respective hydrogel formulation at 25 and 37 °C.

In order to achieve sustained delivery of FL- and HK-cHy-NPs at the desired site of action, the thermoresponsive hydrogel formulation was prepared using our previous NanoSensoGel technology [40]. The developed thermoresponsive hydrogel was present in sol state at room temperature (25 °C), however, at body temperature (37 °C) it converted to gel state. This desired feature was expected to achieve retention of the formulation at the round window membrane and for sustained release of the cHy-NPs. Characteristically, the micellar rearrangement of polymeric units of poloxamer 407 and 188 in the gel had to be responsible for the formation of gel state at higher temperature [49, 50]. The SEM analysis of hydrogels kept at 37 °C before freeze drying showed more arranged honeycomb patterns compared to the hydrogel that was kept at 25 °C (Fig. 8a). These results confirmed the structural changes in the hydrogel morphology related to temperature-associated conformational changes in polymeric backbones. The entrapment of cHy-NPs in the developed hydrogel formulation was further evaluated (Fig. 8b) utilizing the high-resolution focused-emission scanning electron microscopy images for a quantitative and qualitative comparison by artificial intelligence tool. The FL-cHy-NPs and HK-cHy-NPs in the thermoresponsive hydrogels were segmented via a deep learning model created in the DigiM I2S software platform, then particle sizing statistics were computed. Quantification of particle size from 2D images presents a challenge as things such as perspective artifacts, objects occluding others, and low contrast all contribute to reducing the accuracy of segmentation. Size characterization in a 2D image is fundamentally limited by the lack of the 3rd dimension, because of this size distributions are only an estimation. To fully characterize these particles 3D analysis would be needed, using techniques such as Focused Ion Beam Scanning Electron Microscopy (FIB-SEM). The size distribution reported in Table 2 at D10, D50, and D90 were in relative agreement of the TEM image analysis.

**Table 2:** Size distribution analysis at D10, D 50, and D 90

Size Distribution (µm)	D10	D50	D90
HK-cHy-NPs 37 °C	0.26	0.47	0.81
FL-cHy-NPs 37 °C	0.26	0.48	0.75
HK-cHy-NPs 25 °C	0.29	0.51	0.86
FL-cHy-NPs 25 °C	0.10	0.22	0.36

### Release of FL and HK from the hydrogel formulation

The desirable amount of the drugs should be released from the formulation to achieve anticipated pharmacodynamic effect at the targeted site. Therefore, the release study of FL and HK from the respective hydrogel formulation was done at 25 and 37 °C, using PBS (pH 7.4) as the receiving media. At 25 °C, the release of FL and HK was found to be higher as compared to 37 °C. The maximum release of FL and HK from the hydrogel was found to be ~ 50% in one month at 37 °C, however, ~ 60% release of FL and HK was observed in 48 h at 25 °C. (Fig. 8c ‘i’ and ‘ii’). The release kinetics of the formulation was determined by the Korsmeyer-Peppas model (KP-model, Fig. 8c ‘iii’). It was observed that the cumulative percent drug release (CPDR) values fit well with the KP model. The release mechanism values (n) of 25 °C data of FL and HK were found to be between 0.5 and 1 which suggested non-Fickian diffusion mechanism (0.5 < *n* < 1) [51]. However, the ‘n’ values of FL and HK 37 °C release study were found to be < 0.5 which suggested the Fickian release mechanism (Table 3). Since the FL and HK were loaded in the cHy-NPs, the release mechanism primarily depended on the release of FL/HK-cHy-NPs rather than solely on the free FL and HK from the hydrogel formulations. Consequently, the release of cHy-NPs played a crucial role in governing the overall release of FL and HK from the final hydrogel formulation. However, the release mechanism was significantly affected by the environmental temperature because of the thermoresponsive nature of the hydrogel. At 25 °C, the release kinetics follow non-Fickian release because the cHy-NPs diffuse from the gels because of the combination of polymer swelling as well as normal diffusion [52]. However, at 37 °C, the hydrogel formulation was present in gel form. In this case, the swelling effect could be nullified and the NP release mechanism follows the Fickian diffusion kinetics which provides sustained release of the drugs.

**Table 3:** The Korsmeyer-Peppas model fit values of the FL and HK release from the developed formulations

Model parameters	FL-cHy-NPs		HK-cHy-NPs	
	25 °C	37 °C	25 °C	37 °C
K_1_	5.14	2.46	3.37	2.92
n	0.63	0.44	0.73	0.46
R^2^	0.9921	0.9916	0.9976	0.9968

### In vivo ototoxicity prevention efficacy of FL-cHy-NPs and HK-cHy-NPs

**Fig. 9:**
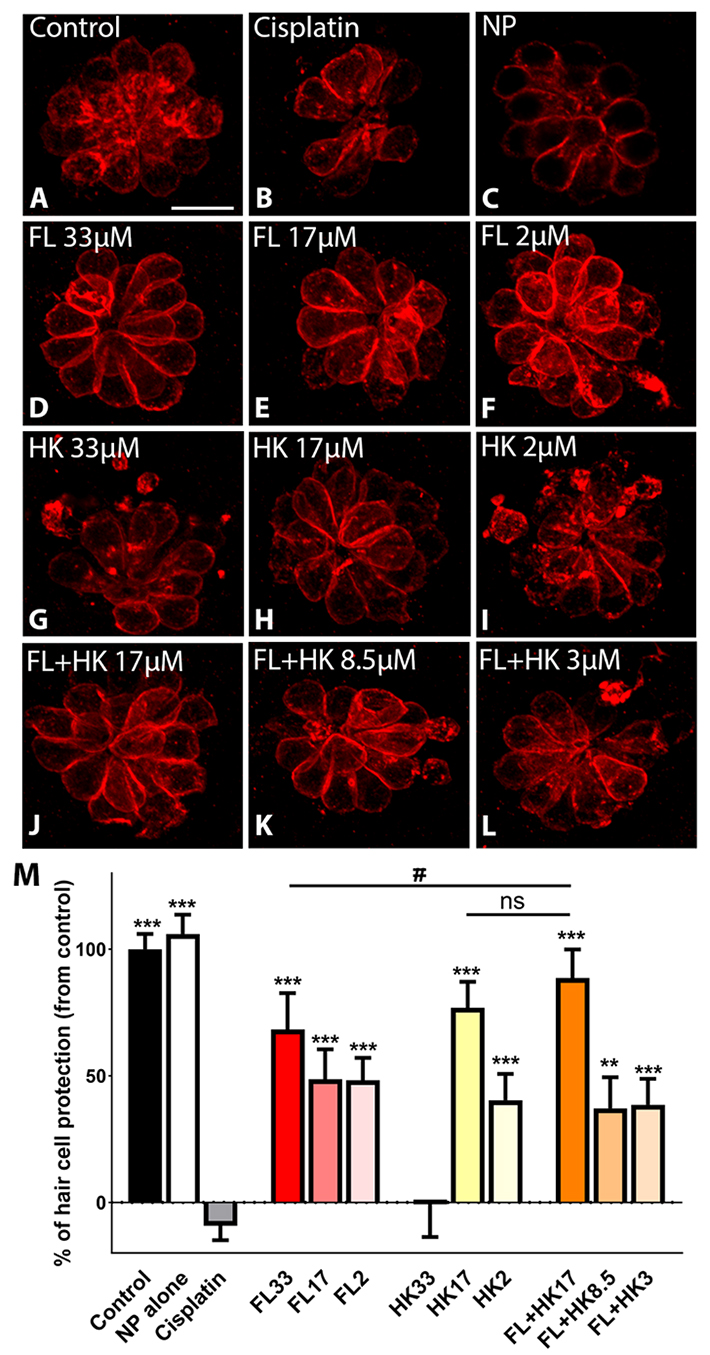
FL and HK protect from cisplatin ototoxicity in zebrafish. **A**-**L**: Representative micrographs of neuromast hair cells immunostained for the hair cell marker, otoferlin (red). **D-F**: FL alone at 33µM, 17µM, or 2µM concentrations. **G-I**: HK alone at 33µM, 17µM, or 2µM concentrations. **J-L**: FL and HK in combination at 17mM, 8.5mM, or 3mM concentrations (each). NP = empty cHy-NPs. Scale bar: 10 μm. **M**: quantification of the number of hair cells per neuromast. Results are expressed as a percentage of protection, with 100% representing control animals and 0% cisplatin-treated fish. A maximum of three neuromasts were inspected per fish were inspected in 10–12 fish. Statistical analysis: One-way ANOVA followed by Dunnett post-test for multiple comparisons. ***p < 0.01 compared to cisplatin alone. #*p* < 0.05 compared to FL + HK17. ns = not significant.

We used zebrafish as our in vivo model to test the therapeutic potential of FL and HK. Figure 9 shows that when fish was incubated with the FL-cHy-NPs and HK-cHy-NPs individually, or in combination, neuromast hair cells were protected against cisplatin-induced ototoxicity. We observed a significant decrease in their numbers in the cisplatin-only group compared to control (Fig. 9B *versus* 9 A). Conversely, treatment with FL, HK, or FL + HK, prevented hair cell loss (Fig. 9D-L). The incubation with empty cHy-NPs (Fig. 9C) did not have any effect. cHy-NPs loaded with FL were protective at the three concentrations tested (Fig. 9D-F and M), while the HK were only beneficial at 17µM and 2µM (Fig. 9G-H and M). The highest HK’s dose (33µM) was toxic to the hair cells (Fig. 9I and M). Finally, we tested a combination of the cHy-NPs (Fig. 9J-M). We found that when fish were co-incubated with cisplatin and the intermediate dose of the cHy-NPs (FL + HK), the protection improved compared to fish incubated with FL or HK at the same dose. These results suggest that FL and HK have the potential to prevent cisplatin-induced hearing loss. Furthermore, the enhanced cytoprotective effect observed with the combination treatment of FL-cHy-NPs and HK-cHy-NPs in the cell studies was mirrored in these in vivo results, where the co-administration of FL and HK provided superior protection compared to the individual treatments. This effect can be attributed to the combined actions of FL and HK in targeting different mechanisms underlying cisplatin-induced ototoxicity, such as ROS detoxification and subsequent inhibition of apoptotic pathways.

## Conclusion

This study discussed the inner-ear targeted sustained drug release crosslinked hybrid nanoparticles (cHy-NPs) embedded in thermoresponsive hydrogel for prophylactic cure and prevention against drug-induced or treatment-induced ototoxicity. The flunarizine and honokiol-loaded cHy-PCDA-PPS-mPEG_2000_-NPs were successfully optimized and studied for long-term stability. The FL-cHy-NPs and HK-cHy-NPs embedded in the thermogel formulation showed sustained release of the FL and HK for 30 days at 37 °C. The standard least square model was found to be a good fit for determining the factors affecting the synthesis of FL-cHy-NPs and HK-cHy-NPs. The good fit of the model for each response was confirmed by ANOVA (*p* < 0.05), Lack of Fit (*p* > 0.05), Actual Vs. Predicted plots, Scatter Index (SIn ≤ 25%), Residual Vs. Actual response plots and Studentized Fit plots. Collectively, the predictive mathematical experimental tool, statistical analysis, AI-assisted deep learning model, in vitro assessments for drug release characteristics, kinetics model, and cell- and animal-based studies have been studied comprehensively to develop a successful novel formulation. These outcomes clearly suggest that the intratympanically delivered crosslinked hybrid nanoformulation developed here may serve as a promising platform to protect the inner ear hair cells not only from the cytotoxic environment of the ototoxic drugs but can also be used for noise-induced and age-related hearing loss. However, these remain to be elucidated in future in vivo investigations to confirm the therapeutic efficacy and translational challenges of the developed novel formulation.

### Electronic supplementary material

Below is the link to the electronic supplementary material.

Supplementary Material 1

## Data Availability

All data generated or analysed during this study are included in this published article [and its supplementary information files] and any additional information are available from the corresponding author on reasonable request..

## Abbreviations

AI: Artificial intelligence
cHy-NPs: Crosslinked hybrid nanoparticles
CCD: Central composite design
CisPt: Cisplatin
CIO: CisPt-induced ototoxicity
DLS: Dynamic light scattering
DoE: Design of experiment
EE: Encapsulation efficiency
FE-SEM: Field emission scanning electron microscopy
FL: Flunarizine
HEI-OC1: House Ear Institute-Organ of Corti 1 cells
HK: Honokiol
HL: Hearing loss
HPLC: High-performance liquid chromatography
Ld: Drug loading
PCDA: 10,12-Pentacosadiynoic acid
PDI: Polydispersity
RMSE: Root Mean Squared Error
ROS: Reactive oxygen species
SIn: Scatter index
STS: Sodium thiosulphate
SEM: Scanning Electron Microscopy
TEM: Transmission electron microscopy
TIHL: Treatment-induced hearing loss
