# Supplementary material for: Crosslinked-hybrid nanoparticle embedded in thermogel for sustained co-delivery to inner ear
Source: J Nanobiotechnology. 2024 Aug 13;22:482. doi: 10.1186/s12951-024-02686-z (PMC11321169; doi:10.1186/s12951-024-02686-z)
Supplement: Supplementary file 1 — Supplementary Material 1 [file 12951_2024_2686_MOESM1_ESM.docx]

**Crosslinked-hybrid nanoparticle embedded in thermogel for sustained co-delivery to inner ear**

Neeraj S. Thakur^1^, Iulia Rus^1^, Aidan Herbert^2^, Marisa Zallocchi^3^, Brototi Chakrabarty^1^, Aditya D. Joshi^1^, Joshua Lomeo^2^, Vibhuti Agrahari^1^*

*Corresponding Author:

Department of Pharmaceutical Sciences, University of Oklahoma Health Sciences Center (OUHSC), 1110 North Stonewall Avenue, Oklahoma City, OK, 73117, USA

[vibhuti-agrahari@ouhsc.edu](mailto:vibhuti-agrahari@ouhsc.edu)

ORCID: Neeraj S. Thakur: 0000-0003-1758-0737

ORCID: Vibhuti Agrahari: 0000-0003-1884-1644

ORCID: Marisa Zallocchi: 0000-0002-6520-988X

**Contents**

[S1. HPLC method development of FL and HK 2](#_Toc161314236)

[S2. Optimization of the synthesis of FL and HK-loaded cHy-NPs using the DoE-CCD approach 6](#_Toc161314237)

[S3. Recipe for 2X blue dye (10 mL) 18](#_Toc161314238)

# S1. HPLC method development of FL and HK

**Table S1. HPLC method parameters for FL and HK qualitative and quantitative analysis**

| **Method parameters** | **FL** | **HK** |
| --- | --- | --- |
| **Mobile Phase (MP)** | A: 0.1%TEA pH 3.5  B: Methanol (100%)  Ratio of A: B = 25:75 | A: ACN: Water (5:4)  B: Methanol (100%)  Ratio of A: B = 45:55 |
| **Column** | C-18 Phenomenex Columbus 5µ, 100×4.8 mm, 25 °C | C-18 Phenomenex Columbus 5µ, 100×4.8 mm, 30 °C |
| **Injection volume** | 10 µL | 10 µL |
| **Flow Rate** | 1 mL/min | 1 mL/min |
| **Wavelength** | 252 nm | 294 nm |
| **Acquisition time** | 4 min | 4 min |


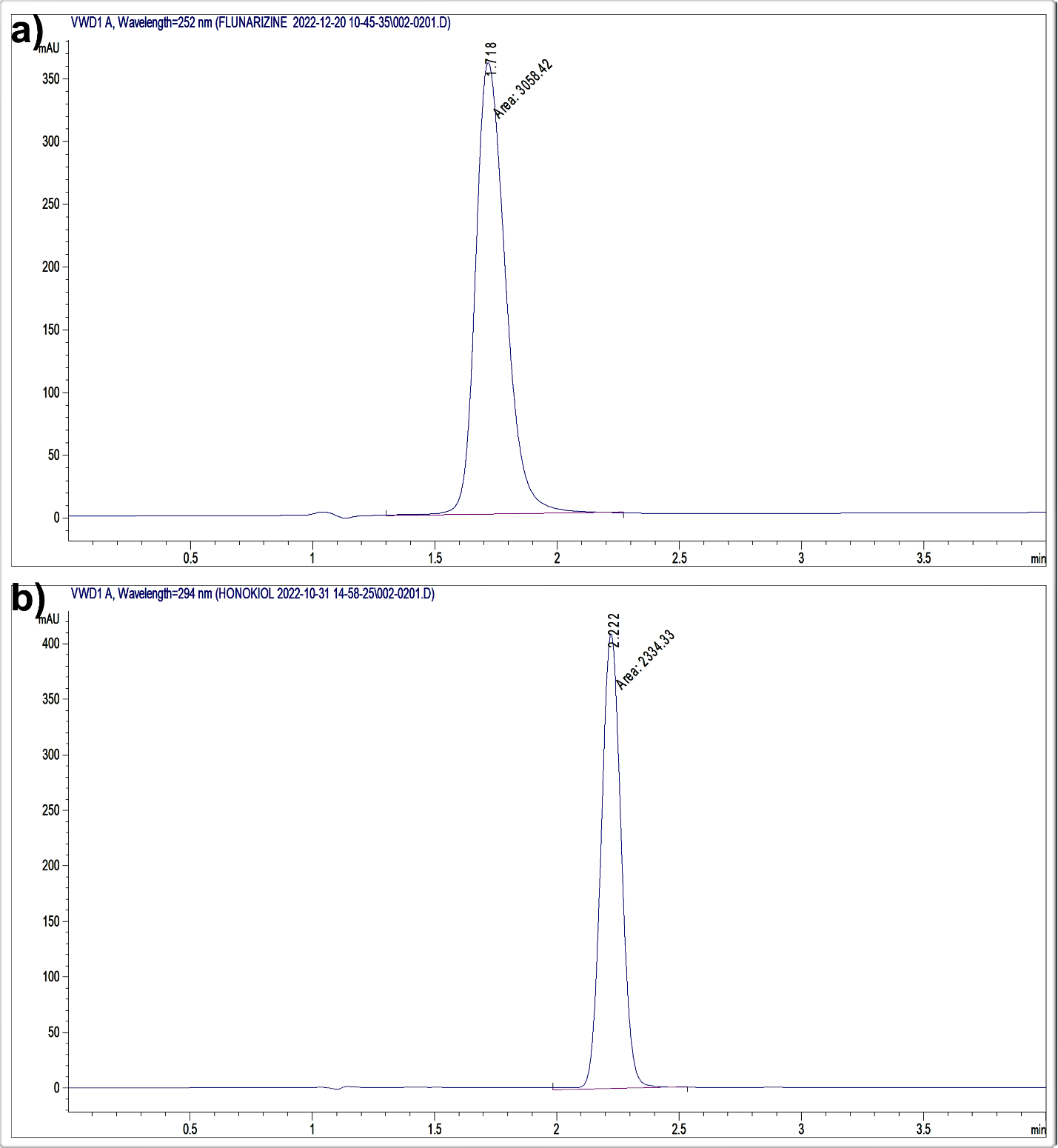


**Figure S1. HPLC chromatogram of (a) Flunarizine and (b) Honokiol**

**Table S2. HPLC validation table for the determination of LOD and LOQ of FL.**

|  | **Symbol** | **FL1** | **FL2** | **FL3** | **Avg** | **SD** |
| --- | --- | --- | --- | --- | --- | --- |
| Slope | S | 33.9095 | 35.51151 | 35.2702 | 34.89705 | 0.863756 |
| Intercept | b | 1.12778 | 1.702778 | 1.82917 | 1.553241 | 0.373842 |
| Number of tests | N | 3 | 3 | 3 |  |  |
| SE of Intercept |  | 0.51769 | 0.378231 | 0.36947 | 0.421796 | 0.083164 |
| SE of Regression | σ | 0.76468 | 0.558685 | 0.54574 | 0.623034 | 0.122842 |
| LOD (µg/mL) | $LOD=\frac{3.3\sigma}{S}$ | 0.07442 | 0.051917 | 0.05106 | **0.059132** | **0.013244** |
| LOQ (µg/mL) | $LOD=\frac{10\sigma}{S}$ | 0.22551 | 0.157325 | 0.15473 | **0.179187** | **0.040135** |
| LOD: Limit of Detection; LOQ: Limit of Quantitation; SE: Standard Error | | | | | | |

**
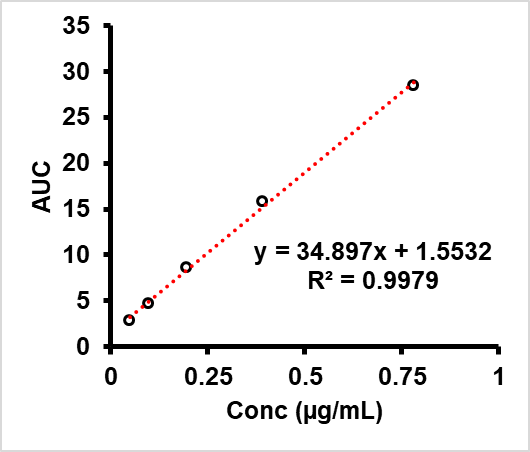
**

**Figure S2: Standard curve for FL quantification**

**Table S3. HPLC validation table for the determination of LOD and LOQ of HK.**

|  | **Symbol** | **H1** | **H2** | **H3** | **Average** | **SD** |
| --- | --- | --- | --- | --- | --- | --- |
| Slope | S | 33.642 | 31.09 | 36.789 | 33.84033 | 2.854672 |
| Intercept | b | 1.6153 | 1.3681 | 1.6028 | 1.528733 | 0.139253 |
| Number of tests | N | 3 | 3 | 3 |  |  |
| SE of Intercept |  | 0.23418 | 0.263024 | 0.298073 | 0.265092 | 0.031997 |
| SE of Regression | σ | 0.345906 | 0.388512 | 0.440283 | 0.391567 | 0.047263 |
| LOD (µg/mL) | $LOD=\frac{3.3\sigma}{S}$ | 0.033931 | 0.041238 | 0.039494 | **0.038221** | **0.003816** |
| LOQ (µg/mL) | $LOD=\frac{10\sigma}{S}$ | 0.10282 | 0.124964 | 0.119678 | **0.115821** | **0.011565** |
| LOD: Limit of Detection; LOQ: Limit of Quantitation; SE: Standard Error | | | | | | |

**
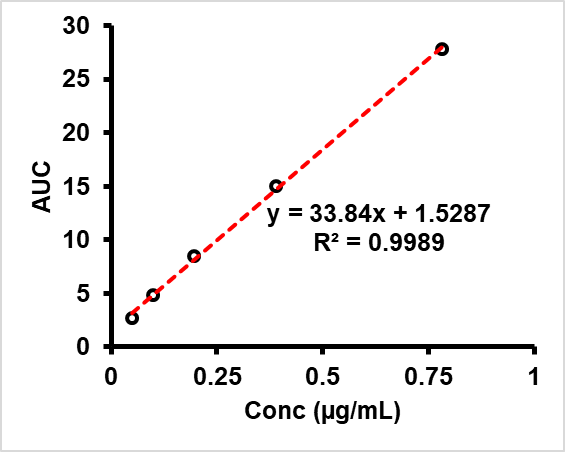
**

**Figure S3: Standard curve for HK quantification**

# S2. Optimization of the synthesis of FL and HK-loaded cHy-NPs using the DoE-CCD approach


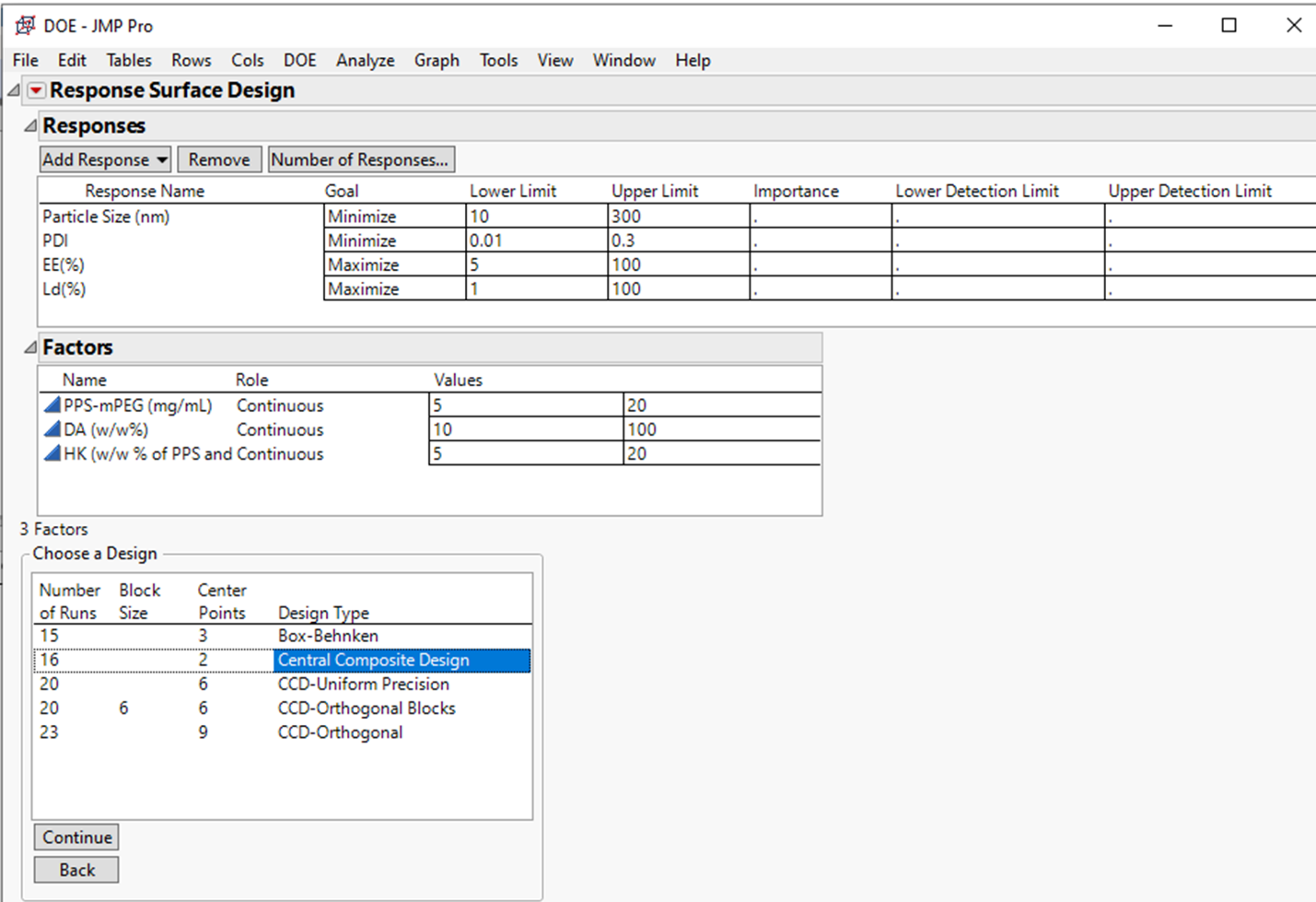


**Figure S4. A DoE window to add responses, and factors, and to choose a design.**

**Figure S5. A DoE window to show how the central composite design was chosen.**


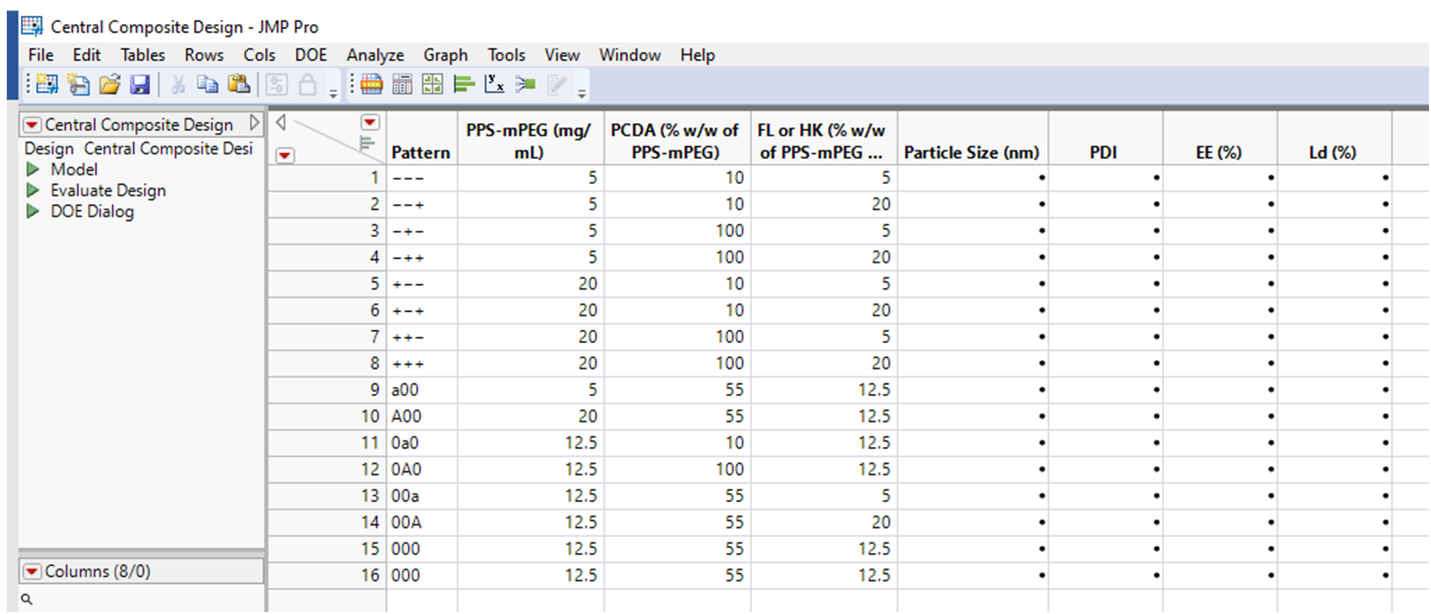


**Figure S6. The central composite design table is generated by the software.**

**Table S4.** The components table shows calculations of the organic phase for all runs to optimize the synthesis of FL- or HK- cHy-PCDA-PPS-PEG NPs using DoE-CCD.

| CCD Factors | | | | | Quantity calculation | | Volume calculation for each run | | | | | |
| --- | --- | --- | --- | --- | --- | --- | --- | --- | --- | --- | --- | --- |
| Run | **Pattern** | **A** | **B** | **C** | **D** | **E** | **F** | **G** | **H** | **I** | **J** | **K** |
|  |  |  |  |  | **[(A×B)/100]** | **[{(A+D) ×C)}/100]** | **A×10** | **D×10** | **E×10** | **E+G+H** | **1000-I** | **I+J** |
| 1 | −−− | 5 | 10 | 5 | 0.50 | 0.28 | 50 | 5.00 | 2.75 | 57.75 | 942.25 | 1000 |
| 2 | −−+ | 5 | 10 | 20 | 0.50 | 1.10 | 50 | 5.00 | 11.00 | 66.00 | 934.00 | 1000 |
| 3 | −+− | 5 | 100 | 5 | 5.00 | 0.50 | 50 | 50.00 | 5.00 | 105.00 | 895.00 | 1000 |
| 4 | −++ | 5 | 100 | 20 | 5.00 | 2.00 | 50 | 50.00 | 20.00 | 120.00 | 880.00 | 1000 |
| 5 | +−− | 20 | 10 | 5 | 2.00 | 1.10 | 200 | 20.00 | 11.00 | 231.00 | 769.00 | 1000 |
| 6 | +−+ | 20 | 10 | 20 | 2.00 | 4.40 | 200 | 20.00 | 44.00 | 264.00 | 736.00 | 1000 |
| 7 | ++− | 20 | 100 | 5 | 20.00 | 2.00 | 200 | 200.00 | 20.00 | 420.00 | 580.00 | 1000 |
| 8 | +++ | 20 | 100 | 20 | 20.00 | 8.00 | 200 | 200.00 | 80.00 | 480.00 | 520.00 | 1000 |
| 9 | a00 | 5 | 55 | 12.5 | 2.75 | 0.97 | 50 | 27.50 | 9.69 | 87.19 | 912.81 | 1000 |
| 10 | A00 | 20 | 55 | 12.5 | 11.00 | 3.88 | 200 | 110.00 | 38.75 | 348.75 | 651.25 | 1000 |
| 11 | 0a0 | 12.5 | 10 | 12.5 | 1.25 | 1.72 | 125 | 12.50 | 17.19 | 154.69 | 845.31 | 1000 |
| 12 | 0A0 | 12.5 | 100 | 12.5 | 12.50 | 3.13 | 125 | 125.00 | 31.25 | 281.25 | 718.75 | 1000 |
| 13 | 00a | 12.5 | 55 | 5 | 6.88 | 0.97 | 125 | 68.75 | 9.69 | 203.44 | 796.56 | 1000 |
| 14 | 00A | 12.5 | 55 | 20 | 6.88 | 3.88 | 125 | 68.75 | 38.75 | 232.50 | 767.50 | 1000 |
| 15 | 000 | 12.5 | 55 | 12.5 | 6.88 | 2.42 | 125 | 68.75 | 24.22 | 217.97 | 782.03 | 1000 |
| 16 | 000 | 12.5 | 55 | 12.5 | 6.88 | 2.42 | 125 | 68.75 | 24.22 | 217.97 | 782.03 | 1000 |
| A: PPS-mPEG (mg/mL)  B: PCDA (w/w % of PPS-mPEG)  C: FL or HK (w/w % of PPS-mPEG & PCDA)  D: PCDA quantity (mg)  E: FL or HK quantity (mg) | | | | | | **F:** PPS-mPEG volume of 100 mg/mL stock (µL) in DCM  **G:** PCDA volume 100 mg/mL stock (µL) in DCM  **H:** FL or HK volume of 100 mg/mL (µL) in DCM  **I:** Volume after addition of F, G, and H in tube (µL)  **J:** Blank solvent (DCM) added (µL)  **K:** Total volume of organic phase (µL) | | | | | | |

**Table S5.** The DoE-CCD table for the synthesis optimization of FL-cHy-PCDA-PPS-NPs

| **Run** | **Pattern** | **PPS-mPEG (mg/mL)** | **DA (w/w%)** | **FL (w/w% of PPS and DA)** | **Particle Size(nm)** | **PDI** | **EE(%)** | **Ld(%)** |
| --- | --- | --- | --- | --- | --- | --- | --- | --- |
| 1 | −−− | 5 | 10 | 5 | 245.6 | 0.107 | 65.37 | 3.27 |
| 2 | −−+ | 5 | 10 | 20 | 253.5 | 0.193 | 46.25 | 9.25 |
| 3 | −+− | 5 | 100 | 5 | 281.1 | 0.285 | 51.60 | 2.58 |
| 4 | −++ | 5 | 100 | 20 | 301.2 | 0.316 | 31.20 | 6.24 |
| 5 | +−− | 20 | 10 | 5 | 220.1 | 0.121 | 66.81 | 3.34 |
| 6 | +−+ | 20 | 10 | 20 | 241.3 | 0.208 | 40.10 | 8.02 |
| 7 | ++− | 20 | 100 | 5 | 310.6 | 0.143 | 79.35 | 3.97 |
| 8 | +++ | 20 | 100 | 20 | 325.2 | 0.222 | 46.30 | 9.26 |
| 9 | a00 | 5 | 55 | 12.5 | 293.1 | 0.266 | 26.80 | 3.35 |
| 10 | A00 | 20 | 55 | 12.5 | 281.3 | 0.169 | 89.34 | 11.17 |
| 11 | 0a0 | 12.5 | 10 | 12.5 | 215.2 | 0.173 | 63.56 | 7.95 |
| 12 | 0A0 | 12.5 | 100 | 12.5 | 279.7 | 0.232 | 62.13 | 7.77 |
| 13 | 00a | 12.5 | 55 | 5 | 246.8 | 0.158 | 91.37 | 4.57 |
| 14 | 00A | 12.5 | 55 | 20 | 263.2 | 0.238 | 61.37 | 12.27 |
| 15 | 000 | 12.5 | 55 | 12.5 | 245.7 | 0.179 | 83.25 | 10.41 |
| 16 | 000 | 12.5 | 55 | 12.5 | 242.2 | 0.167 | 85.61 | 10.70 |

**Table S6.** The DoE-CCD table for the synthesis optimization of HK-cHy-PCDA-PPS-NPs

| **Run** | **Pattern** | **PPS-mPEG (mg/mL)** | **PCDA (w/w%)** | **HK (w/w% of PPS and DA)** | **Particle Size(nm)** | **PDI** | **EE(%)** | **Ld(%)** |
| --- | --- | --- | --- | --- | --- | --- | --- | --- |
| 1 | −−− | 5 | 10 | 5 | 233.4 | 0.197 | 51.33 | 2.567 |
| 2 | −−+ | 5 | 10 | 20 | 245.7 | 0.203 | 31.91 | 6.382 |
| 3 | −+− | 5 | 100 | 5 | 283.3 | 0.305 | 64.1 | 3.205 |
| 4 | −++ | 5 | 100 | 20 | 317.6 | 0.36 | 23.6 | 4.72 |
| 5 | +−− | 20 | 10 | 5 | 211.8 | 0.139 | 63.66 | 3.183 |
| 6 | +−+ | 20 | 10 | 20 | 226.6 | 0.206 | 36.3 | 7.26 |
| 7 | ++− | 20 | 100 | 5 | 301.4 | 0.254 | 91.26 | 4.563 |
| 8 | +++ | 20 | 100 | 20 | 285.1 | 0.287 | 59.36 | 11.87 |
| 9 | a00 | 5 | 55 | 12.5 | 282.2 | 0.258 | 32.89 | 4.111 |
| 10 | A00 | 20 | 55 | 12.5 | 296.5 | 0.209 | 97.4 | 12.18 |
| 11 | 0a0 | 12.5 | 10 | 12.5 | 225.6 | 0.192 | 59.33 | 7.416 |
| 12 | 0A0 | 12.5 | 100 | 12.5 | 287.7 | 0.282 | 85.13 | 10.64 |
| 13 | 00a | 12.5 | 55 | 5 | 241.7 | 0.108 | 88.63 | 4.432 |
| 14 | 00A | 12.5 | 55 | 20 | 251.7 | 0.188 | 42.87 | 8.574 |
| 15 | 000 | 12.5 | 55 | 12.5 | 275.7 | 0.182 | 88.69 | 11.09 |
| 16 | 000 | 12.5 | 55 | 12.5 | 262.3 | 0.159 | 86.37 | 10.8 |


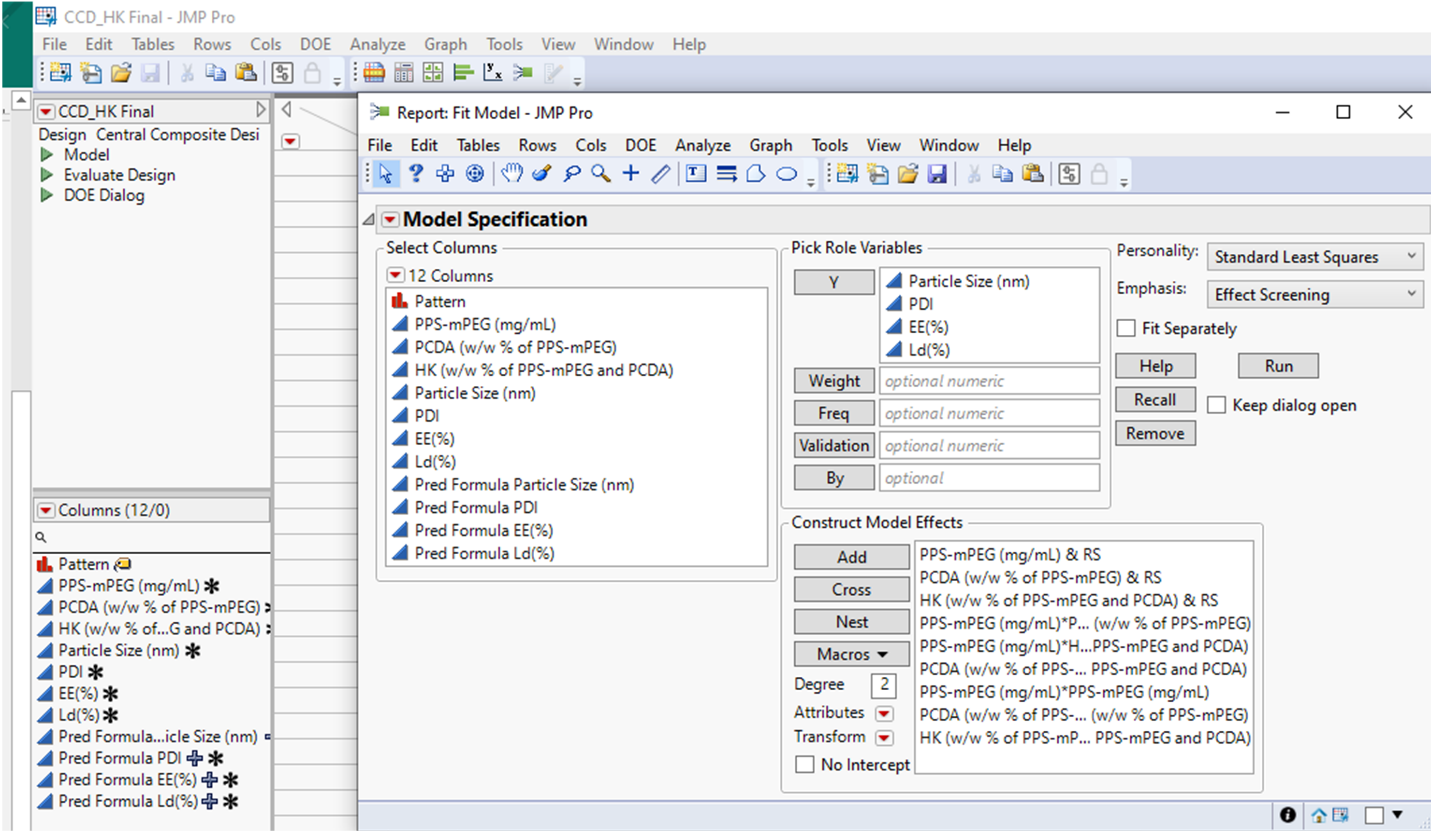


**Figure S7. A DoE-CCD window to show how the model was applied after putting the response data.**


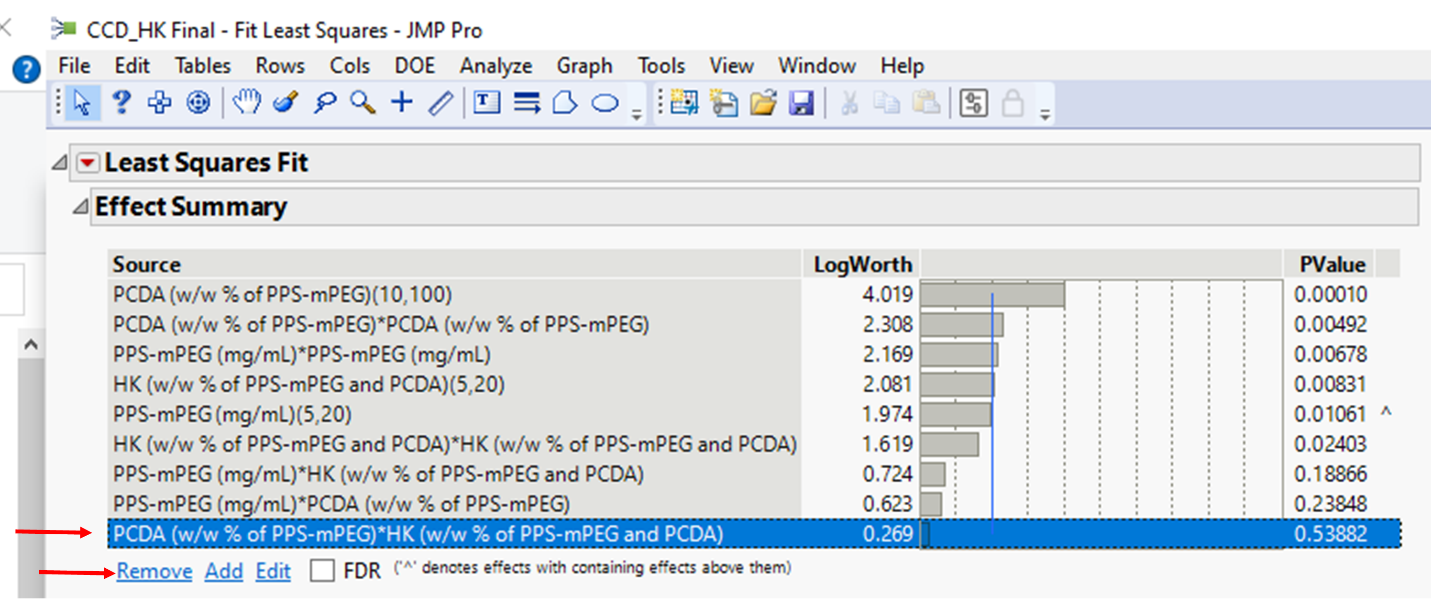


**Figure S8. A DoE-Fit least squares window to show how the nonsignificant factors were deleted.**


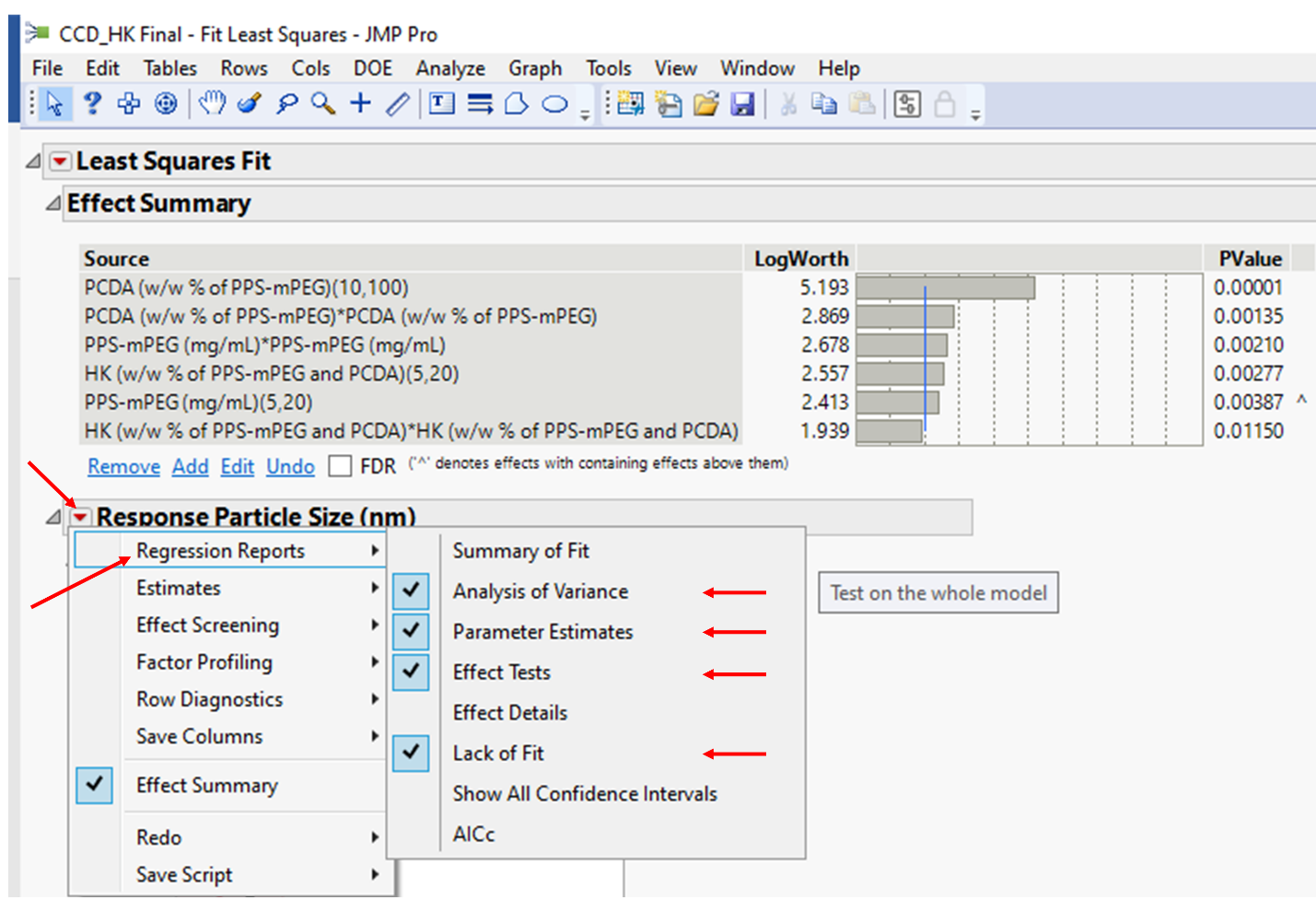


**Figure S9. A DoE-Fit least squares window to show how the regression reports were generated.**


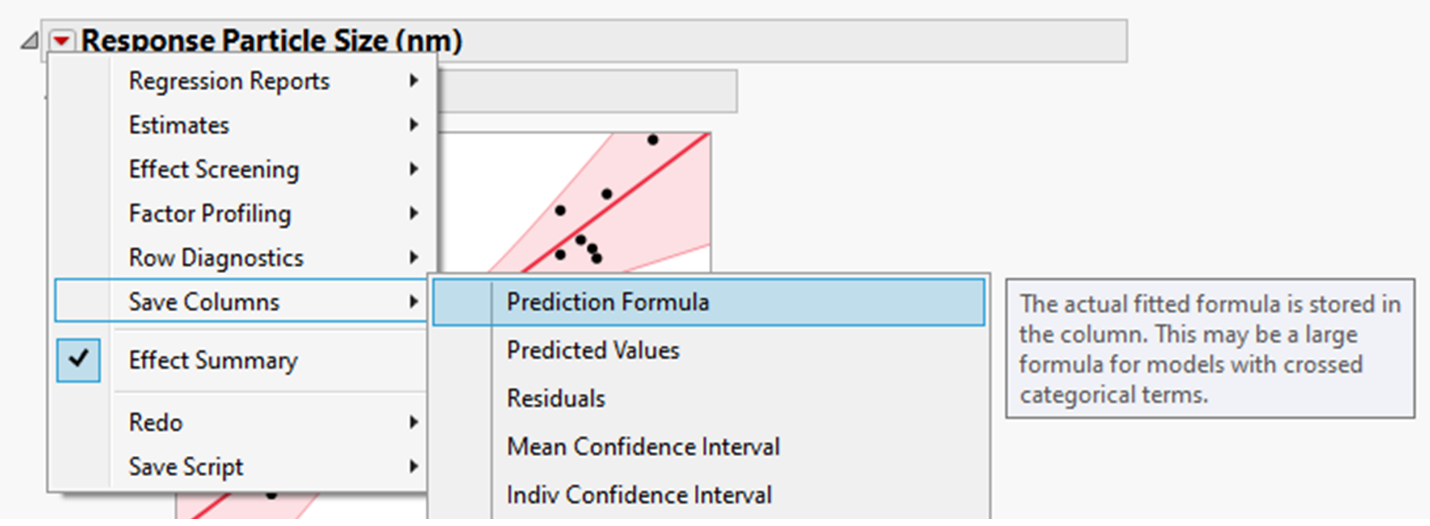


**Figure 10. A DoE-Fit least squares window to show how the prediction formula was added to the DoE-CCD table.**


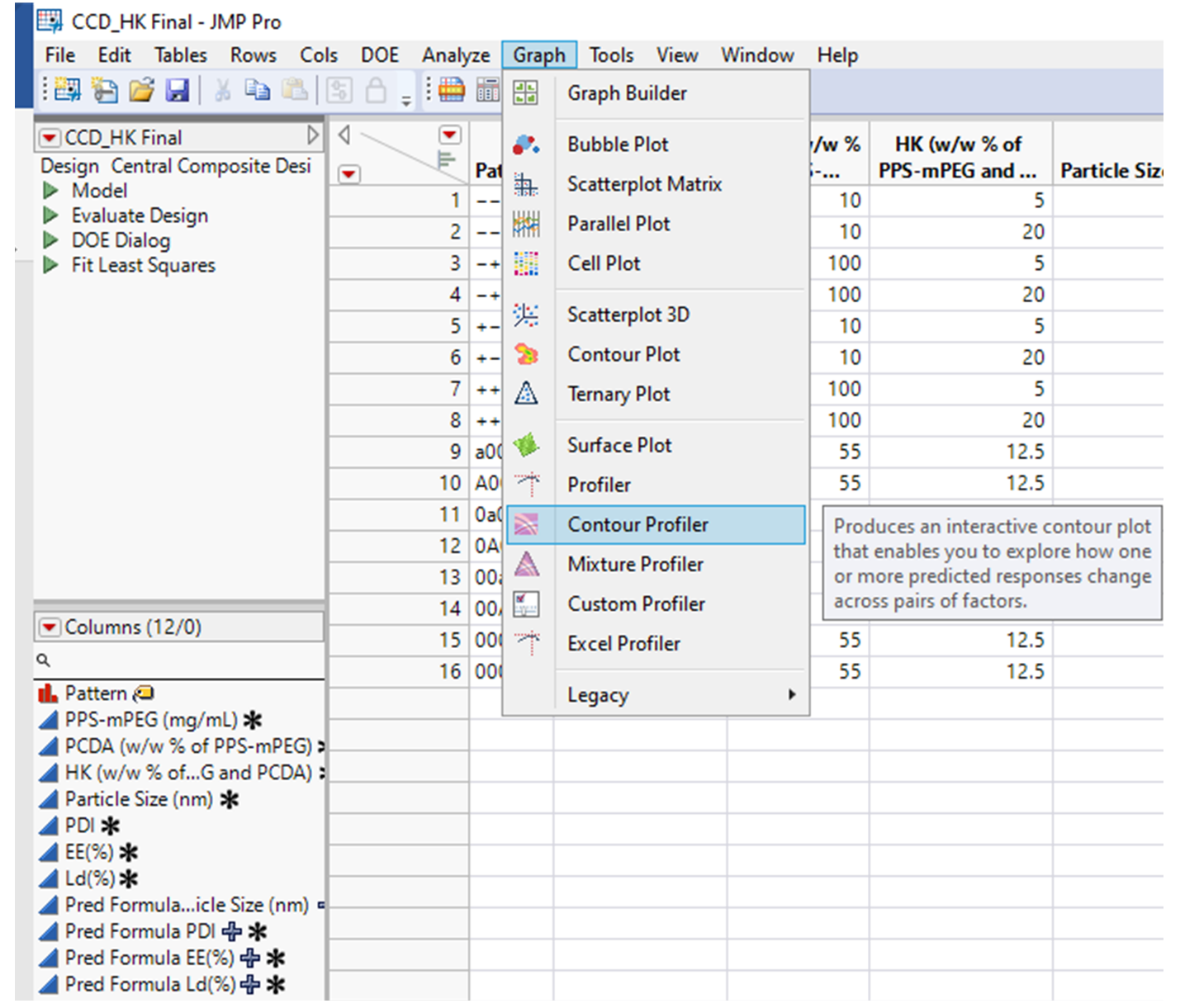


**Figure S11. A DoE-CCD window to show how the contour profiler window was accessed.**


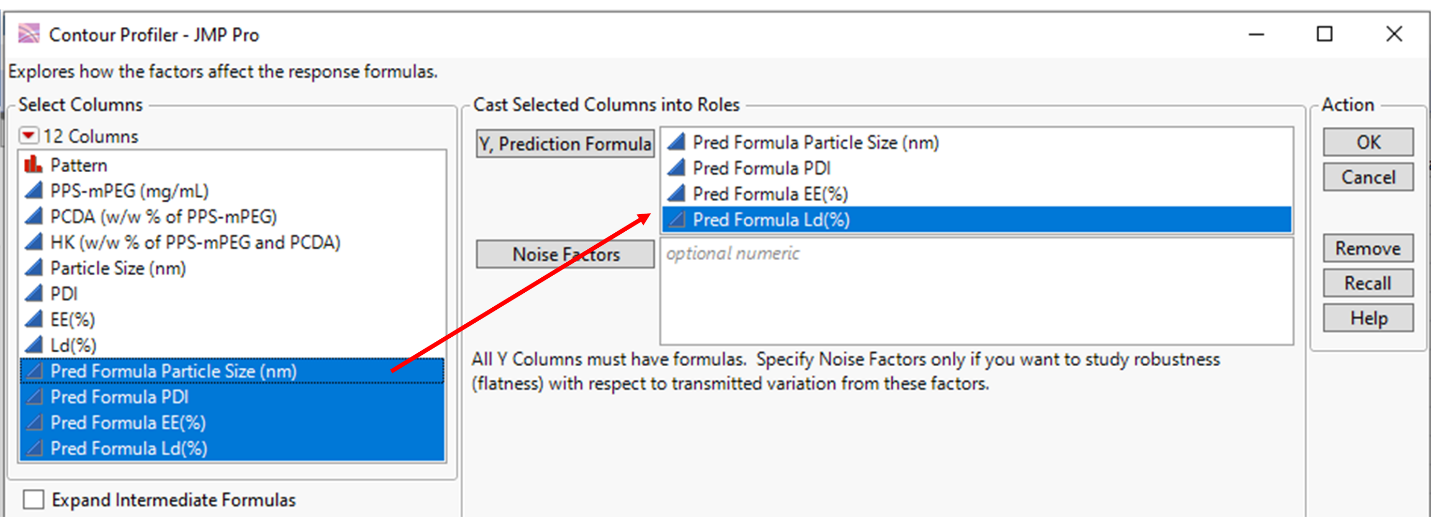


**Figure S12. A DoE-Fit least squares window to show how the contour profilers were generated.**


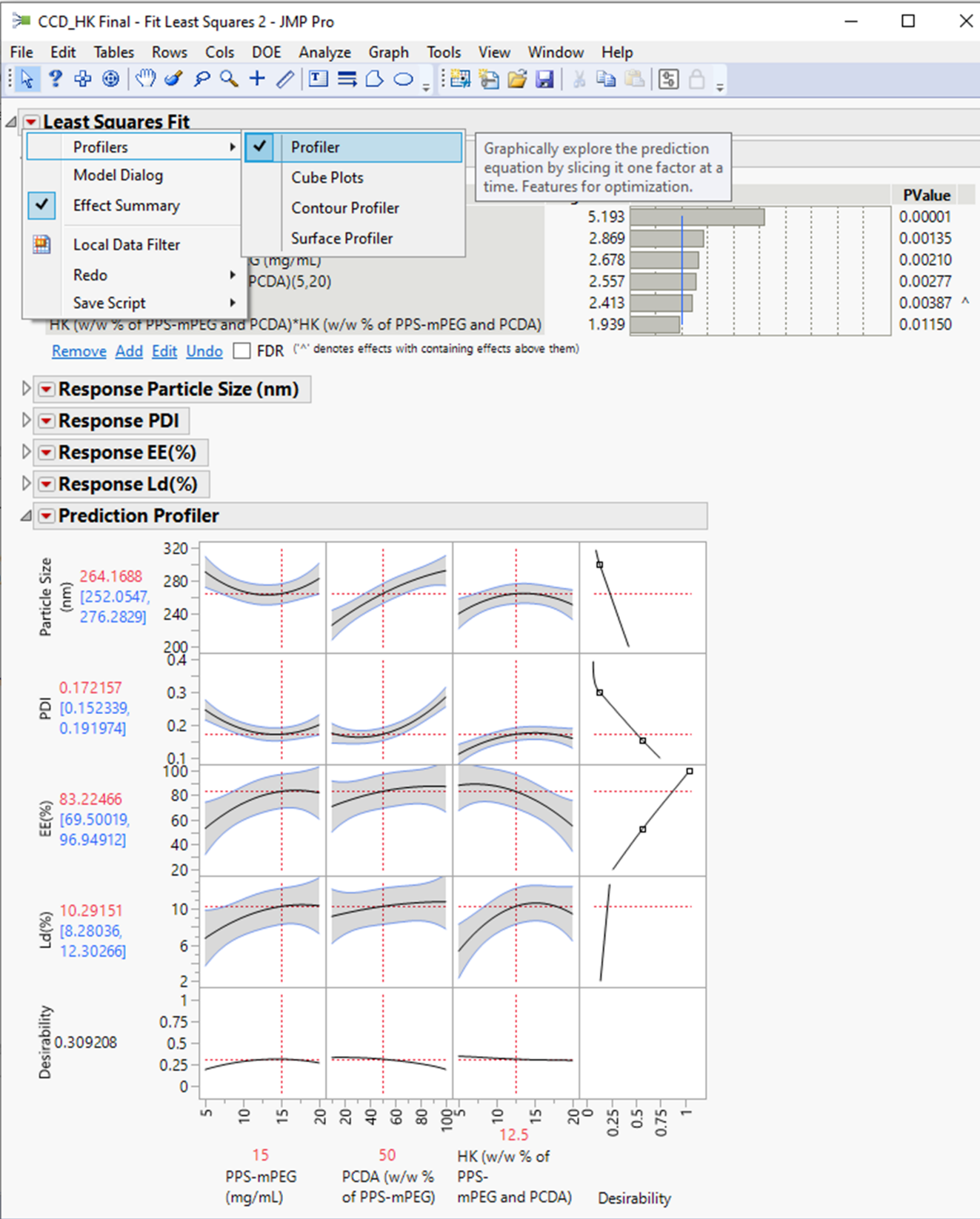


**Figure S13. A DoE-Fit least squares window to show how the prediction profiler graphs were generated.**


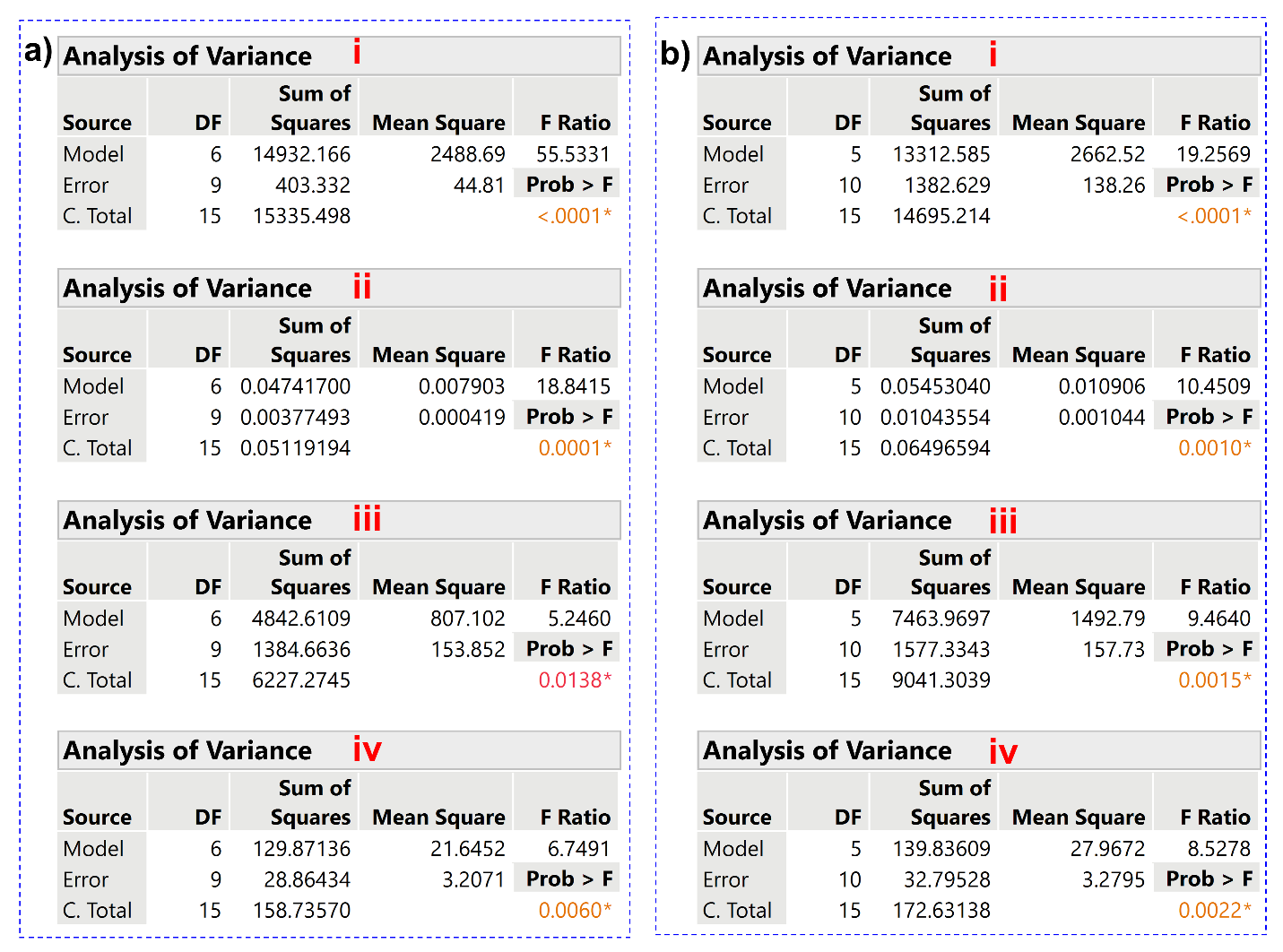


**Figure S14**. Analysis of variance (ANOVA) analysis tables after running the linear fit model for the optimization of the synthesis of (a) FL-cHy-NPs, and (b) HK-cHy-NPs. The responses for each NP preparation presented as ‘i’ Particle Size (nm), ‘ii’ PDI, ‘iii’ EE (%), and ‘iv’ Ld (%). In each table, the *F* value of each response is ≤0.05 suggesting that the ANOVA is significant, and the model is a good fit.


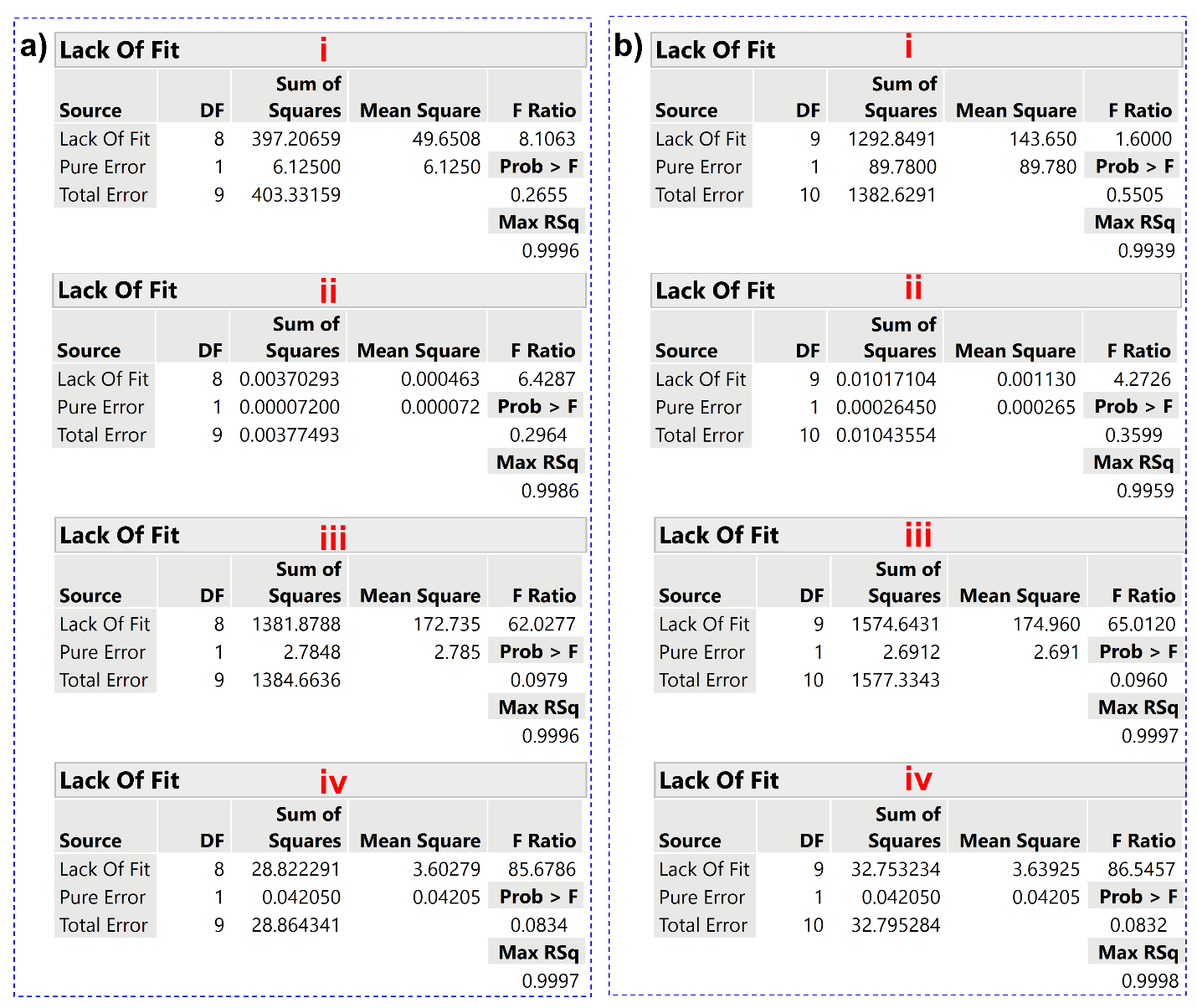


**Figure S15**. Lack of fit analysis tables after running the linear fit model for the optimization of the synthesis of (a) FL-cHy-NPs, and (b) HK-cHy-NPs. The responses for each NP preparation presented as ‘i’ Particle Size (nm), ‘ii’ PDI, ‘iii’ EE (%), and ‘iv’ Ld (%). In each table, the *F* value of each response is >0.05 suggesting that the lack of fit is not significant, and the model is a good fit.


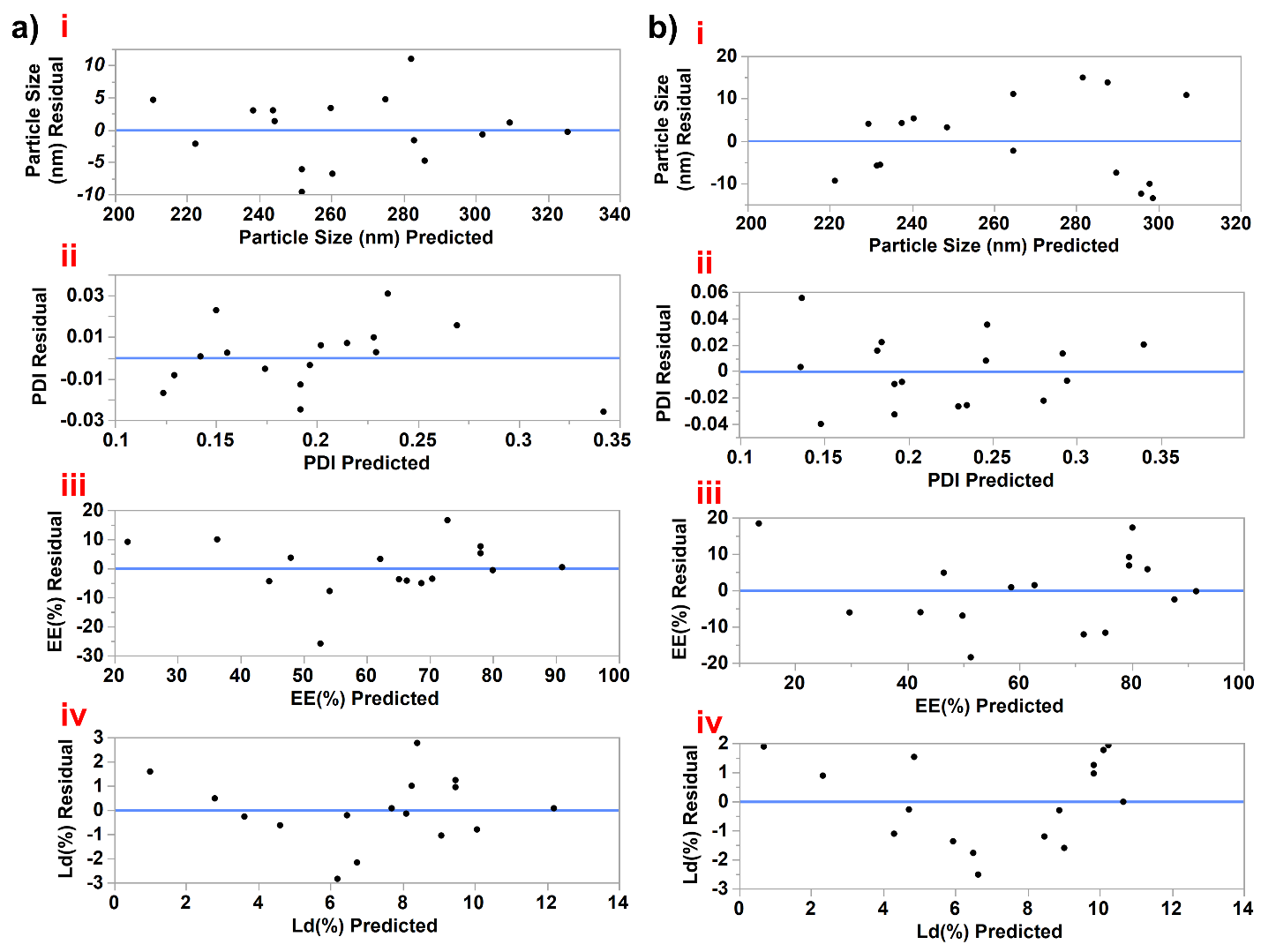


**Figure S16.** The residual vs predicted plots of each response after running the linear fit model for the optimization of the synthesis of (a) FL-cHy-NPs, and (b) HK-cHy-NPs. The responses for each NP preparation presented as ‘i’ Particle Size (nm), ‘ii’ PDI, ‘iii’ EE (%), and ‘iv’ Ld (%).


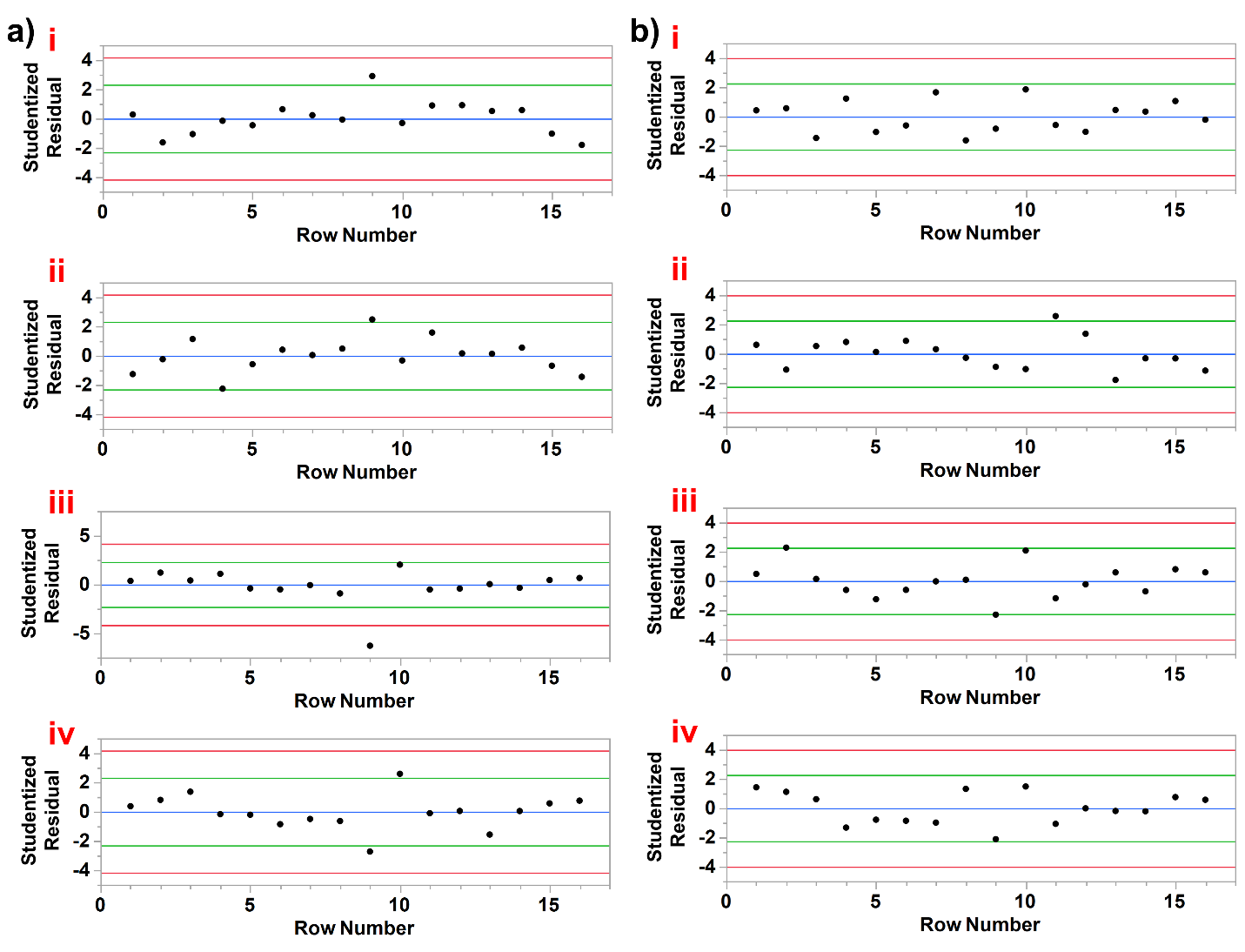


**Figure S17.** The studentized residual fit plots of each response after running the linear fit model for the optimization of the synthesis of (a) FL-cHy-NPs, and (b) HK-cHy-NPs. The responses for each NP preparation presented as ‘i’ Particle Size (nm), ‘ii’ PDI, ‘iii’ EE (%), and ‘iv’ Ld (%). In graphs, the red lines showing the 95% simultaneous limits (Bonferroni test) and green line showing the individual limit. Here, the studentized residuals were calculated as dividing the regression model residual by adjusted standard error using JMP software. All studentized residuals were present within the 95% limits, except row 9 of EE (%) response (iii), in FL-cHy-NPs showed outlier effect. All responses showed good fit of the Standard Least Square model including the EE (%) response (iii), in FL-cHy-NPs, therefore, the outlier (run 9) was not deleted.

# S3. Recipe for 2X blue dye (10 mL)

1. 0.5 M Tris HCl pH 6.8 (2.5 mL)
2. 10% SDS (4 mL)
3. Glycerol (2 mL)
4. 0.1% Bromophenol Blue (0.3 mL)
5. 2- mercaptoethanol (1 mL)
6. Deionized water )0.2 mL)
